# Frontotemporal lobar degeneration changes neuronal beta-frequency dynamics during the mismatch negativity response

**DOI:** 10.1016/j.nicl.2024.103671

**Published:** 2024-09-10

**Authors:** Alistair Perry, Laura E. Hughes, Natalie E. Adams, Michelle Naessens, Niels A. Kloosterman, Matthew A. Rouse, Alexander G. Murley, Duncan Street, P. Simon Jones, James B. Rowe

**Affiliations:** a MRC Cognition and Brain Sciences Unit, University of Cambridge, Cambridge, United Kingdom; b Department of Clinical Neurosciences and Cambridge University Hospitals NHS Trust, University of Cambridge, United Kingdom; c Institut für Psychologie I, Universität zu Lübeck, Germany; d Max Planck Institute for Human Development, Berlin, Germany

**Keywords:** Neurophysiology, Frontotemporal lobar degeneration, Beta-band, Magnetoencephalography, Time-frequency, Mismatch negativity

## Abstract

The consequences of frontotemporal lobar degeneration include changes in prefrontal cortical neurophysiology, with abnormalities of neural dynamics reported in the beta frequency range (14–30 Hz) that correlate with functional severity. We examined beta dynamics in two clinical syndromes associated with frontotemporal lobar degeneration: the behavioral variant of frontotemporal dementia (bvFTD) and progressive supranuclear palsy (PSP). Whilst these two syndromes are partially convergent in cognitive effects, they differ in disease mechanisms such as molecular pathologies and prefrontal atrophy. Whether bvFTD and PSP also differ in neurophysiology remains to be fully investigated. We compared magnetoencephalography from 20 controls, 23 people with bvFTD and 21 people with PSP (Richardson’s syndrome) during an auditory roving oddball paradigm. We measured changes in low and high total beta power responses (14–22 and 22–30 Hz respectively) over frontotemporal cortex in the period of the mismatch negativity response (100–250 ms post-stimulus). In controls, we found increased 14–22 Hz beta power following unexpected sensory events (i.e. increased deviant versus standard response), from right prefrontal cortex. Relative to controls, PSP reversed the mismatch response in this time–frequency window, reflecting reduced responses to the deviant stimuli (relative to standard stimuli). Abnormal beta at baseline in PSP could account for the reduced task-modulation of beta. Across bvFTD and PSP groups, the beta response to deviant stimuli (relative to standard stimuli) correlated with clinical severity, but not with atrophy of the prefrontal source region. These findings confirm the proposed importance of higher-order cortical regions, and their beta-power generators, in sensory change detection and context-updating during oddball paradigms. The physiological effects are proposed to result from changes in synaptic density, cortical neurotransmitters and subcortical connections, rather than merely atrophy. Beta-power changes may assist clinical stratification and provide intermediate outcomes for experimental medicine studies of novel therapeutic strategies.

## Highlights

• We confirmed a unique role for the R IFG during the roving oddball paradigm.
• R IFG showed increased beta1 power change to unexpected deviant stimuli.
• Progressive Supranuclear Palsy selectively impaired these beta dynamics in R IFG.
• This was not observed in behavioral variant of frontotemporal dementia.
• Clinical severity, but not cortical atrophy, was associated with beta power change.

## 1 Introduction

Frontotemporal lobar degeneration (FTLD) encompasses a diverse set of molecular pathologies and spectrum of clinical disorders. These include the behavioural variant of frontotemporal dementia (bvFTD) (Rascovsky et al., 2011) and progressive supranuclear palsy (PSP) (Höglinger et al., 2017). They change cognition and behaviour by diverse mechanisms, including the loss of cells (atrophy), synapses (Holland et al., 2020, Malpetti et al., 2022), and neurotransmitters in frontotemporal cortex and their subcortical connections (Boeve et al., 2022, Ferrer, 1999, Gazzina et al., 2019, Grossman et al., 2023). These mechanisms contribute to the neurophysiological abnormalities associated with bvFTD and PSP (Samudra et al., 2024), observed by electro- and magnetoencephalography (MEG). Of particular interest are the changes to neural dynamics in the beta frequency range (14–30 Hz), as both groups exhibit abnormalities in both local power and connectivity during rest (Sami et al., 2018) and in task-states (Adams et al., 2021, Hughes et al., 2018, Hughes and Rowe, 2013). Moreover, these changes in beta-dynamics correlate with clinical severity (Cope et al., 2017, Hughes et al., 2018).

bvFTD and PSP have much in common in their neurophysiology, and cognitive and behavioural deficits (Ghosh et al., 2012, Murley et al., 2020a, Murley et al., 2020b, Ranasinghe et al., 2016). There is also commonality in their deficits of major neurotransmitters including GABA and glutamate, that are reduced in both PSP and bvFTD (Adams et al., 2021, Ferrer, 1999; Foster et al., 2000, Levy et al., 1995; Murley et al., 2020b, Murley and Rowe, 2018), including from prefrontal cortex (Murley et al., 2020b). However, there are also marked differences in their underlying molecular pathology (Boeve et al., 2022, Gazzina et al., 2019, Perry et al., 2017) and degree of atrophy (Grossman et al., 2023). For instance, unlike bvFTD, PSP has relatively preserved volume/thickness of the lateral prefrontal cortex (Ghosh et al., 2012, Perry et al., 2022, Whitwell et al., 2020), and this may lead to differences in some aspects of prefrontal neurophysiology.

There is convergent evidence for impairment of beta-dynamics in people with frontotemporal lobar degeneration. For example, in roving auditory oddball paradigms, both bvFTD and PSP show diminished “mismatch” responses, indicative of error signals generated in response to unexpected deviant tone stimuli (relative to regular standard tones that develop strong predictions) (Adams et al., 2021, Cope et al., 2022, Hughes et al., 2013, Shaw et al., 2021). Again, prefrontal responses are particularly affected in these syndromes, along with impaired beta-mediated feedback signalling from these areas (Adams et al., 2021, Cope et al., 2022, Hughes et al., 2013). Degeneration of prefrontal areas in another FTLD-associated syndrome, nonfluent variant primary progressive aphasia, also changes frontotemporal beta connectivity (Cope et al., 2017). Lastly, beta-band abnormalities extend to resting-state oscillations (Sami et al., 2018) and response inhibition paradigms (Hughes et al., 2018).

Frequency-specific brain dynamics have been linked to normative cognitive processes (Buzsaki and Draguhn, 2004, Engel et al., 2001). Beta dynamics, for example, are critical for sensory change detection and feedback signalling (Arnal and Giraud, 2012, Bastos et al., 2012, Haenschel et al., 2000, Meindertsma et al., 2018); an asymmetry of feedforward vs. feedback signalling in hierarchical cortical networks is widely adopted with lower-frequency feedback signals (i.e. beta/alpha) generated from higher-level regions such as prefrontal cortices, and higher-frequency (i.e. gamma) feedforward signals propagating back up from sensory areas. Low beta frequencies (beta1, <22 Hz) and high beta frequencies (beta2, >22 Hz) may actually have distinct roles in context signalling (Engel and Fries, 2010) and change detection (Haenschel et al., 2000). However, in the context of the roving oddball paradigm and its mismatch negativity response, it remains unknown whether the frontotemporal generators differ in their total beta-band activity. It is important to note at the outset that power in the beta frequencies is not only driven by neural oscillations. Indeed, much of the observed beta power arises from non-oscillatory transient responses that are local features of simultaneous currents up- and down-cortical columns with distinct durations (Jones, 2016, Sherman et al., 2016).

Here, we aimed to determine whether people with bvFTD and PSP show similar or distinct disturbances of beta responses. We used a roving auditory oddball paradigm to probe beta dynamics, induced in response to expected (i.e. standard) or deviant stimuli. The paradigm is well-tolerated in clinical populations and is sensitive to bvFTD, PSP and their pharmacological manipulations. In frontotemporal regions, we quantify beta power differences following sensory change (following *deviant* versus *standard* responses). Our hypothesis was that prefrontal regions (that are high in a frontotemporal information processing hierarchy) manifest disease-related loss of beta modulation, whereas temporal regions lower in the hierarchy do not. As right prefrontal responses were selectively increased in controls during unexpected events (relative to repeated tones), we then focus on this area to study beta differences between bvFTD and PSP, and the moderation of cortical neurophysiology by atrophy.

## 2 Methods

### 2.1 Participants and neuropsychological assessment

Fifty people with probable frontotemporal lobar degeneration (26 bvFTD, 24 PSP-Richardson’s syndrome) and 20 age-/sex-matched healthy adults underwent magnetoencephalography in two independent studies (Adams et al., 2021, Perry et al., 2022). Five patients (3 bvFTD, 2 PSP) took part in both studies and the magnetoencephalography session closest to the MRI session date was used. Hence, the remaining number of patients included in analysis prior to quality assurance was 23 bvFTD and 22 PSP persons.

People with bvFTD and PSP were recruited with a clinical diagnosis of probable bvFTD, with or without parkinsonism (Rascovsky et al., 2011) or probable PSP-Richardson’s syndrome (PSP-RS) (Höglinger et al., 2017). At the time of study recruitment, these probable diagnoses represented definitive clinical diagnoses (without postmortem confirmation), according to the international consensus diagnostic criteria for each disease group*.* Patients who had clinically presented initially with “PSP-Frontal” phenotype had subsequently converted to Richardson’s syndrome (Grimm et al., 2019). The participants had no other history of significant neurological or psychiatric illnesses. Written informed consent was given in accordance with the 1991 Declaration of Helsinki, and the studies were approved by the local research ethics committee. After quality control review, one participant with PSP was excluded because they had half the average number of trials after artifact rejection.

A subset of bvFTD and PSP individuals were on medications during the study (SI Table 1).

Neuropsychological assessment included the Addenbrookes Cognitive Examination-revised (ACE-R) (Mioshi et al., 2006), Frontal Assessment Battery (FAB) (Dubois et al., 2000), Graded Naming Test (Mckenna and Warrington, 1983), INECO Frontal Screening Test (Torralva et al., 2009) and Hayling Sentence Completion test (Burgess and Shallice, 1996). The Cambridge Behavioural Inventory (CBI-R) (Wear et al., 2008) was completed by a close informant for people with bvFTD and PSP. We also derived patients’ disinhibition phenotype score from the CBI-R subscales of *abnormal behaviour*, *stereotypic movement and behaviour* and *eating* (Hughes et al., 2018).

The Frontotemporal Dementia Rating Scale (FRS) (Mioshi et al., 2010) and Progressive Supranuclear Palsy Rating Scale (PSPRS) (Golbe and Ohman-Strickland, 2007) were used as measures of clinical severity for people with bvFTD and PSP respectively. To pool them together, we z-normalized clinical scores within each group, after inversion of z-scores for FRS (so that higher scores on ‘FRS’ and PSPRS both indicated more severe disease).

### 2.2 Magnetoencephalography, preprocessing and source localisation

Participants had magnetoencephalography recorded during a passive roving auditory oddball paradigm (for full details of paradigm see (Adams et al., 2020) and Supplementary Information 1.1). The paradigm involved a series of repeated tones (*rep_n_*) which changed pseudorandomly in frequency (i.e. a deviant tone) after 3–10 repetitions (approximate Poisson distribution). The paradigm was performed with eyes-open in three blocks of five minutes, while participants watched a silent movie. Passive movie watching was included to improve the consistency of eye movements across participants, and reduce the likelihood of individuals falling asleep, maintaining similar attention levels. Participants were also under continuous video monitoring to ensure none fell asleep, and they were not asked to attend to the auditory stimuli. The number of tone presentations in the paradigm were adjusted from the first (*M* = 1822.13, *SD* = 90.48) to second study (*M* = 1577.23, *SD* = 95.25), to accommodate other resting and task paradigms acquired in the scanning session. After artefact rejection, participants averaged 1669 (*SD* = 151) trials per session used in time–frequency analysis.

A magnetically-shielded room (IMEDCO) and Elekta VectorView (Elekta Neuromag, Helsinki) were used to record magnetoencephalography, with 306-channel recordings (planar gradiometer pair and magnetometer at each site), sampled at 1000 Hz. Electro-occulography (EOG) and 3D digitized fiducial and scalp points were also recorded.

Preprocessing was performed as in previous studies (Adams et al., 2022, Perry et al., 2022) (available at https://github.com/AlistairPerry/FTLDMEGTF) using SPM12 (v7771), FieldTrip (Oostenveld et al., 2011) and OSL (https://github.com/OHBA-analysis/osl-core) software in Matlab (2019a). Briefly, processing included (1) MaxFiltering with signal-space separation (v2.2.12, Elekta Neuromag; to remove noisy signals and head motion correction), (2) downsampling (500 Hz), (3) notch-filtering (removing 45–55 Hz and 95–105 Hz), (4) bad channel detection (using *osl_detect_artefacts*), and lastly (5) independent components analysis (ICA). ICA was performed with *fastica* (https://research.ics.aalto.fi/ica/fastica) and custom code (https://github.com/MRC-CBU/riksneurotools/blob/master/MEEG/detect_ICA_artefacts.m) to remove eye-blink and movement components that were correlated with the EOG time series and spatial artifact templates*.* After trial epoching (see below), bad trials were then removed (again using OSL’s *osl_detect_artefacts*) to remove remaining artifacts (e.g. motor).

Two separate epoching procedures were performed. For time–frequency estimation, single-trial data were padded to include both the preceding and following trial of each event-of-interest (−750 to 1050 ms) to enable robust time–frequency estimation. For analysis of evoked mismatch waveforms (Perry et al., 2022), data were epoched in the original range (−100 to 400 ms), bad trials were removed, condition-averaged (with robust averaging), low-pass filtered (45 Hz), and then baseline corrected (−100 to 0 ms). For full acquisition and preprocessing details see Supplementary information 1.2.

Source-localisation was performed on both the single-trial data (i.e. padded data) and condition-averaged magnetoencephalography signals, using all channels. This was performed in SPM12 using a single shell cortical mesh, estimated from individual T1-weighted image (co-registered using fiducial and head points). The T1w-volumetric images used either MPRAGE (Adams et al., 2021) or 7T MP2RAGE T1w-MRI for co-registration (see below for acquisition and processing details) (Perry et al., 2022). The source signals were estimated using COH inversion (Pascual-Marqui, 2002), and waveforms extracted from literature-derived MNI coordinates of bi-lateral cortical “mismatch negativity” sources (Adams et al., 2020, Garrido et al., 2008): Auditory cortex (L AUD, [−42, –22, 7]; R AUD, [46, −14, 8]), superior temporal gyrus (L STG, [−61, –32, 8]; R STG, [59, −25, 8]), and inferior frontal gyrus (L IFG, [−46, 20, 8]; R IFG, [46, 20, 8]) (SI Fig. 1A). The local peak was identified within a 7 mm radius and extracted to form pseudo-local field potential (LFP) responses.

We chose the COH method for comparability with our previous oddball studies in these diseases (Adams et al., 2021, Cope et al., 2022, Kocagoncu et al., 2022, Phillips et al., 2016). All inversion accuracies indicated a strong fit of the reconstructed signals to the sensor-level data, as all participants had variance explained greater than 75 % (*M* = 95.33, *SD* = 2.81). Moreover, the source locations have been validated by intracranial locations during the same paradigm (Phillips et al., 2016). Alternative methods such as multiple sparse priors (MSP) could be more optimal for deriving focal signal responses (and thus with higher within-subject model evidence). Whilst the MSP places constraints on the signals extracted, we nonetheless find a high correspondence between signals derived from the two methods. Across all study participants, the average correlation for evoked signals (across the entire *peri*-stimulus period) from the right auditory cortex is 0.73 for standard trials (*rep6*, *SD* = 0.20) and 0.71 (*SD* = 0.23) for deviants (dev). Reconstruction performance in example participants from each group are illustrated in SI Fig. 1B, and show the MMN coordinates to considerably overlap with maximum activation in these individuals.

For single-trial data, we focused on the deviant condition (*dev*) and sixth repetition (*rep6*) of the same frequency tone (c.f. a locally standard stimulus), to estimate the change in total time–frequency beta power to an unexpected sensory event (i.e. *dev*).

For the condition-averaged data we further calculated a difference waveform from the *dev* and *rep6* (*rep6*-*dev*) responses, to derive the mismatch response (MMN). The mean MMN was calculated from the averaged mismatch response between 125 and 175 ms post-stimulus presentation. This window overlaps with the peak of the mismatch response in independent studies (Garrido et al., 2009).

### 2.3 Time-frequency estimation

**Fig. 1:**
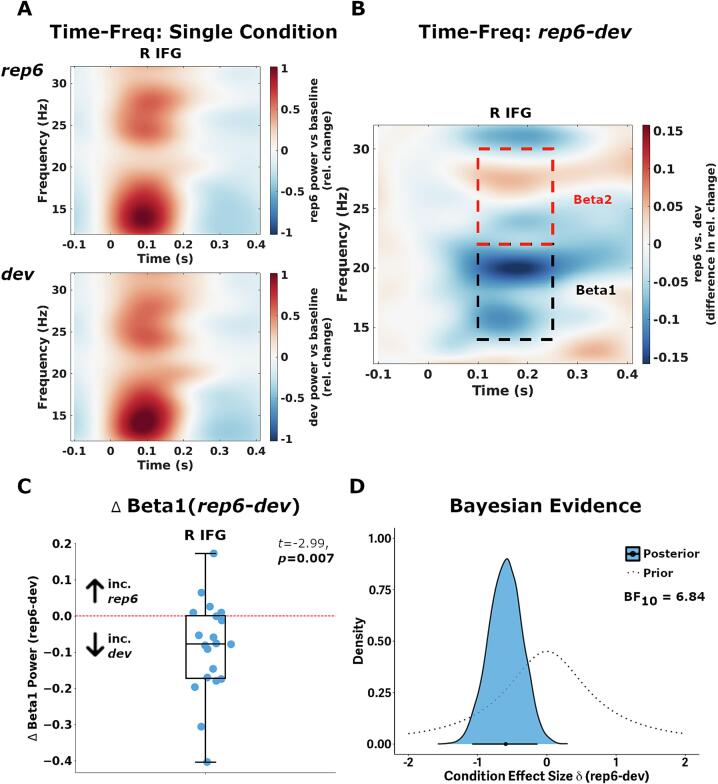
Schematic of analysis workflow for estimating beta time–frequency power and its modulation in controls during the roving oddball paradigm. A) Responses during *rep6* and *dev* conditions are used to estimate time–frequency responses (top and bottom panels respectively), measured by relative power compared to baseline period (−100 to 0 ms). Shown is the average control group response for each condition in the right inferior frontal gyrus region (R IFG). B) For each participant the difference in power between the two conditions is then calculated, with the average difference defined within *a priori* low (beta1, 14–22 Hz; black window) and high-beta (beta2, 22–40 Hz; red) windows. Shown here is the average condition-difference map in the right inferior frontal gyrus region (R IFG), with heatmap values interpolated (c.f. https://eelkespaak.nl/blog/customizing-common-m-eeg-plots-part-2-the-time-frequency-representation-tfr). C) Distribution of each control participants condition-dependent changes in beta1 power. Box plot represents group mean with interquartile range of 25 % and 75 % percentile, and whiskers indicating 95 % probability density. D) Bayesian modelling of posterior distributions (shaded blue) for evidence of condition effect size under the alternative hypothesis, along with the prior distribution (cauchy, λ = 0.707; dotted opaque curve). Thick horizontal line indicates 95 % highest posterior density interval (HPDI). (For interpretation of the references to colour in this figure legend, the reader is referred to the web version of this article.)

We used a sliding window Fourier transform (Mitra and Pesaran, 1999) (step size, 50 ms; window size, 500 ms; frequency resolution, 2 Hz) to calculate time–frequency representations of total magnetoencephalography power for each trial in the *dev* and *rep6* conditions (Fig. 1A; here shown group average trial responses), across all frontotemporal sources. We used a single Hanning taper in FieldTrip for the range 12–32 Hz (4 Hz smoothing box), as this decomposition is optimal for estimating lower-frequency responses. For each trial, time–frequency power was extracted from the standard epoch of −100 to 400 ms, and then averaged over condition. Power modulation at a given time-point and frequency for each condition is calculated as change relative to the average baseline power (−100 to 0 ms) for each frequency bin (i.e. using ‘relchange’ in *ft_freqbaseline*). We then calculated the power difference between the *dev* and *rep6* conditions (*rep6-dev*) (Fig. 1B). We focus on beta responses in the 100–250 ms window in which the classic peak “mismatch response” is generated and which previously have shown alterations in PSP and bvFTD (Cope et al., 2022, Garrido et al., 2008, Hughes et al., 2013, Phillips et al., 2015). As lower (beta1, 14–22 Hz) and higher (beta2, 22–30 Hz) beta frequencies show differential sensory change responses (Haenschel et al., 2000), our principal analyses focused upon mean power difference (i.e. *rep6*-*dev*) within temporal windows 100–250 ms and separately for frequencies 14–22 (beta1) and 22–30 Hz (beta2) (Fig. 1B).

To dissociate the phase-locked responses to stimulus presentation (Donner and Siegel, 2011), we computed evoked time frequency responses again after averaging the padded single-trial data. The average baseline power used above for total power responses (i.e. derived from single-trial estimates) was used to baseline correct the evoked responses.

### 2.4 MR imaging

Participants completed a T1-weighted structural scan at 7T (Siemens TERRA, Erlangen, Germany) or 3T (Siemens PRIMSA). Acquisition parameters were at 7T MP2RAGE, 32-channel headcoil, 0.75 mm isotropic voxels, TE = 1.99 ms, TR = 4300 ms, and inversion times = 840 ms/2370 ms.; and at 3T MPRAGE 1.1 mm isotropic voxels TE = 2.9 ms, TR = 2000 ms. For all parameters see (Perry et al., 2022) and Supplementary information 1.3.

For patients with 7T imaging (13 bvFTD, and 15 PSP), we tested whether atrophy in the right inferior frontal gyrus correlated with beta power responses, and differential responses between bvFTD and PSP. Following (Perry et al., 2022), we first calculated right inferior frontal gyrus grey-matter volume (GMV) using an anatomical mask that overlapped with the magnetoencephalography source region (github.com/inm7/jubrain-anatomy-toolbox, and https://neurovault.org/images/776918) (Eickhoff et al., 2005) (Fig. 4A). We chose this mask given the inherent smoothness of atrophy disease patterns in both bvFTD and PSP, and our observations that the MEG sphere is centrally located amongst the anatomical regions the mask is derived from. 7T T1 images were bias regularised (threshold of 0.0001), tissue-segmented (SPM12 v7771), and normalized to MNI space via diffeomorphic registration (i.e. DARTEL) (Ashburner, 2007). The normalization included modulation in order to preserve local volume but without spatial smoothing. GMV was calculated by the total sum of GM voxel probabilities enclosed within the anatomical mask.

Again only for those with 7T imaging, we also tested grey matter volume differences between control (n = 18), bvFTD and PSP groups elsewhere using whole-brain voxel-based morphometry (VBM) (Ashburner and Friston, 2000) (Supplementary Information 1.4). Normalized images for VBM (only) were spatially smoothed using an 8 mm full-width at half-maximum (FWHM) kernel.

### 2.5 Statistical analysis

#### 2.5.1 Beta responses by condition and group

For the control group, we performed paired *t*-tests (two-tailed) in controls to determine the functional regions with significant condition-differences in beta1 (14–22 Hz; 100–250 ms) and beta2 (22 Hz–30 Hz) responses, across *dev* and *rep6* trial conditions. Given 6 frontotemporal sources, we controlled for multiple comparisons by using Bonferroni-corrected α of 0.0083 (0.05/6 regions). We also performed exploratory cluster-based analysis of power differences across *dev* and *rep6* conditions across the whole time–frequency space (i.e. 0–400 ms, 14–30 Hz). Using FieldTrip (*ft_freqstatistics*), cluster significance was determined using monte-carlo permutations (*n* = 5000) with critical α = 0.025 (two-tailed).

For group comparisons, we focussed on the right inferior frontal gyrus, that had shown condition differences in beta responses in the control group. We performed one-way ANOVA (bvFTD, PSP, controls) to detect group differences in condition-power change (i.e. *rep6*-*dev*). Sidak correction was used for post-hoc contrasts.

#### 2.5.2 Associations with atrophy and disease severity

We performed Pearson correlational analyses to test for associations of beta power change with cortical atrophy (of right inferior frontal gyrus) and disease severity. Severity was quantified in terms of cognition (ACE-R and FAB), the disinhibition subscale of the CBI, and disease-specific functional rating scales (FRS, PSPRS).

As control analyses, we additionally performed ANCOVAs to determine whether age confounded group differences; and whether baseline beta power influenced the effect of condition and group on beta responses to unexpected sensory events.

Descriptive frequentist statistics were performed in Python/R and accompanying Bayesian tests were also conducted (in JASP for Bayes Factors, https://jasp-stats.org/; and “BayesFactor” R package for density estimates). For Bayesian tests, high Bayes Factors (BF_10_) indicate moderate (>3) or strong (>10) evidence for the alternate hypothesis (i.e. that there is a difference or a correlation), while low Bayes Factors indicate < 0.33 (moderate) and < 0.10 (strong) evidence in favour of the null hypothesis (i.e. that there is no difference, or correlation).

All preprocessing, time–frequency decomposition, and analysis scripts are available at https://github.com/AlistairPerry/FTLDMEGTF.

## 3 Results

People with bvFTD or PSP did not differ from controls in both age and sex, while bvFTD were younger than PSP *(p*_sidak_ = 0.049, BF_10_ = 3.60, Table 1)*.* As expected, bvFTD and PSP were impaired in MMSE, all ACE-R subscales, INECO, FAB, and the Hayling tests. People with bvFTD were relatively impaired to PSP on the INECO and were reported to have more severe behavioural impairments by global CBI-R and its disinhibition score. The majority of CBI-R sub scores also revealed greater behavioural impairments in bvFTD (SI Table 2).

**Table 1:** Demographic and neuropsychological information of study participants.

	CON	bvFTD	PSP	CON vs. bvFTD	CON vs. PSP	bvFTD vs. PSP
	M (SD)			*p*-val (Bayes Factor)		
*n*	20	23	21			
Sex	M15:F5	M19:F4	M13:F8	n.s	n.s	n.s
Age	66.75 (4.79)	62.95 (7.98)	69.19 (8.51)	n.s (1.14)	n.s (0.51)	* (3.36)

*Neuropsych*						
MMSE	29.65 (0.49)	23.43 (7.79)	26.38 (3.19)	** (29.94)	*** (371.99)	n.s (0.78)
ACE-R						
Total	96.6 (2.48)	69.62 (23.73)	78.86 (10.1)	*** (2504.76)	*** (2.65e + 4)	n.s (0.95)
Attention	17.9 (0.31)	14.43 (5.1)	16.71 (2.08)	* (7.76)	* (3.50)	n.s (1.06)
Memory	24.65 (1.46)	17.1 (8.29)	21.19 (3.75)	*** (158.73)	** (63.90)	n.s (2.03)
Verbal Fluency	12.6 (1.6)	4.52 (3.46)	5.1 (2.88)	*** (1.37e + 9)	*** (3.75e + 9)	n.s (0.35)
Language	25.65 (0.81)	21.24 (6.05)	23.71 (1.79)	** (22.69)	*** (268.61)	n.s (1.37)
Visuospatial	15.8 (0.41)	12.33 (3.8)	12.14 (3.8)	*** (85.81)	*** (188.52)	n.s (0.31)
INECO Total	25.2 (2.2)	10.89 (6.96)	17.58 (4.7)	*** (4.60e + 7)	*** (108.95e + 3)	** (23.95)
FAB Total	17.2 (1.06)	9.76 (5.19)	12.62 (3.07)	*** (24.78e + 3)	*** (57.18e + 3)	n.s (1.35)
Hayling	5.85 (0.93)	2.06 (1.39)	3.5 (2.16)	*** (4.43e + 8)	*** (291.23)	n.s (2.60)

*p < 0.05; **p < 0.01; ***p < 0.001, uncorrected.

bvFTD, behavioural variant frontotemporal dementia; CON, controls; PSP, progressive supranuclear palsy; BF, Bayes Factor; Conventional thresholds for Bayes Factors represent substantial (>3), strong (>10) and very strong (>30) evidence in favour of alternate hypothesis.

ACE-R, Addenbrooke's Cognitive Examination-Revised; FAB, frontal assessment battery; MMSE mini-mental state exam.

The groups with bvFTD and PSP did not show significantly different number of trials removed from the oddball paradigm (SI Table 3). Owing to their characteristically reduced ocular activity (Bologna et al., 2009), the PSP group reported significantly decreased blink rate and also number of bad ICA components removed (SI Table 3). No group differences were identified between bvFTD and PSP.

### 3.1 Modulation in beta power by condition in controls

All frontotemporal sources showed increased beta power in the *dev* and *rep6* conditions, relative to baseline (R IFG shown in Fig. 1A, all other regions SI Fig. 3; see SI Fig. 2 for condition-averaged evoked waveforms). The right inferior frontal region (R IFG) was the only source region to show significant condition-dependent modulation in total beta1 power, with a relative increase in *dev* trials (*t* = −2.99, *p* = 0.0075, BF_10_ = 6.54; 95 % HPDI = [−1.08, −0.13], Fig. 1C and D). All other regions show moderate-to-strong evidence for no condition difference (*p* > 0.46, BF = 0.23–0.30; SI Fig. 4). Exploratory cluster-based analysis (two-tailed) across the whole time–frequency window (14–30 Hz, 0–400 ms), revealed no significant cluster indicating condition difference in the right inferior frontal region (*p*_FWE_ > 0.285).

No region showed differences (surviving Bonferroni correction) between *dev* and *rep6* conditions in the beta2 frequency range *(p*_unc_ *>* 0.025, BF_10_ = 0.26–2.48). To differentiate evoked responses (phase-locked to stimulus onset) from total (both phase-locked and non-phase-locked) beta1 dynamics, we re-ran time–frequency estimates *after* averaging the single-trial data (for evoked condition-difference maps in the R IFG see SI Fig. 5). No significant condition modulation in evoked beta1 power was found in the right inferior frontal gyrus (*p*_unc_ = 0.14, BF_10_ = 0.66), nor any other source region (*p*_unc_ > 0.26, BF_10_ = 0.25–0.42).

### 3.2 Group differences in beta modulation to sensory change

**Fig. 2:**
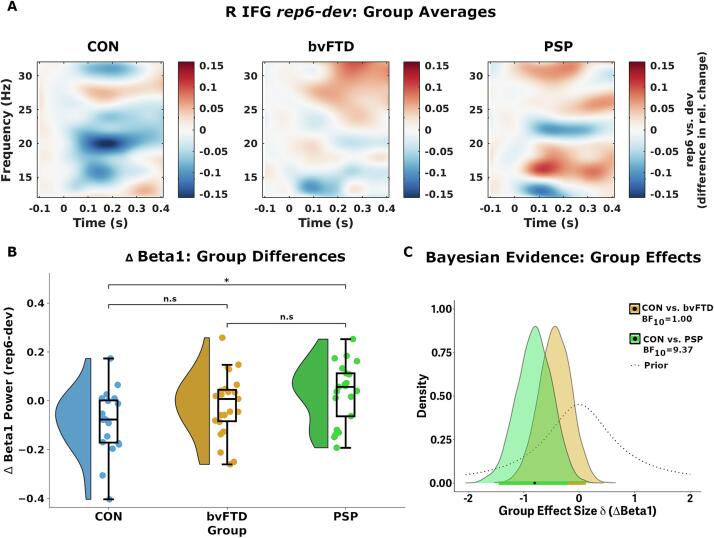
Group differences in low-frequency modulation during the roving oddball paradigm. A) Group averages in low-frequency modulation (*rep6*-*dev*) across the entire estimated time–frequency space in the right inferior frontal region (R IFG), for controls (left panel), bvFTD (middle) and PSP (right) persons. B) Group distributions of beta1 condition-difference scores in the R IFG region. Boxes represents group mean with interquartile range of 25 % and 75 % percentile, and whiskers indicating 95 % probability density. C) Bayesian modelling of posterior distributions for evidence of a group effect between controls and bvFTD (orange shading) and PSP (green) persons under the alternative hypothesis (two-tailed), with the prior distribution shown in the dotted opaque curve. Thick horizontal lines indicate 95 % highest posterior density intervals (HPDI). (For interpretation of the references to colour in this figure legend, the reader is referred to the web version of this article.)

Given the condition-dependent change in beta power in the right inferior frontal source and in the beta1 range, our remaining analyses focused here. For control and patient group-average low-frequency modulation between the *dev* and *rep6* conditions see Fig. 2A. ANOVA indicated group differences in beta1 modulation between *dev* and *rep6* conditions in right inferior frontal cortex (*F*[2,66] = 4.12, *p* = 0.014, BF_10_ = 3.55) (Fig. 2B). Post hoc comparisons reveal this effect to be driven by differences between control and PSP groups (*t* = −3.01, *p*_sidak_ = 0.013, BF_10_ = 9.38; 95 % HPDI = [−1.46, −0.19]; Fig. 2B, and for Bayesian posterior estimates see Fig. 2C). In contrast to controls, people with PSP on average show decreased beta power to *dev* (relative to *rep6*) (see also SI Fig. 6A). People with bvFTD showed no significant difference to either control (*t* = 1.77, *p*_sidak_ = 0.242, BF_10_ = 1.00, 95 % HPDI = [−1.02, 0.12]) or PSP groups (*t* = −1.32, *F*[2,60] = 4.58, *p*_sidak_ = 0.466, BF_10_ = 0.61), yet paired *t-*tests indicate no significant difference between the *dev* and *rep6* conditions (*t* = −0.68, *p* = 0.504, BF_10_ = 0.27) within this disease group.

Post hoc inspection yields the dynamics of stimulus repetition-dependent beta changes in PSP (SI Fig. 6). While controls show a gradual decrease in beta1 power with increasing tone repetitions, PSP and bvFTD do not (SI Fig. 6A), consistent with a failure of short-term plasticity in the context of neurodegeneration.

Auxiliary analyses indicated group differences in beta1 power remain after adjustment for age (ANCOVA; *F*[2,60] = 4.58, *p* = 0.014). Group effects also remain if analyses are adjusted for baseline deviant power (*F*[2,60] = 4.58, *p* = 0.014), while noting the increase in (log-normalized) baseline beta power1 in people with PSP compared to bvFTD (*t* = −2.87, *p*_sidak_ = 0.013, BF_10_ = 7.61) (SI Fig. 6B). Moreover, group differences are also robust to controlling for the relative number of bad trials removed for each individual (*F*[2,60] = 5.77, *p* = 0.005). Analysis of classical mismatch waveforms and MMN responses in right inferior frontal cortex indicates no group differences (*F*[2,61] = 0.40, *p* = 0.673, BF_10_ = 0.17) (SI Fig. 7).

### 3.3 Auxiliary analysis: Baseline period

Given the rapid-event design of the oddball paradigm we employed a baseline period of 100 ms so that our estimates of baseline beta activity are not influenced by the preceding trial activity. To determine our effects are robust to a baseline capturing the conventional 2–3 cycles of frequency activity (i.e. for the slowest beta at 14 Hz), we additionally computed time–frequency estimates with a baseline of −150 to 0 ms. We find that our within-group effects of condition-dependent beta1 modulation in controls are robust to using a longer baseline (*t* = −2.85, *p*_unc_ = 0.011, BF_10_ = 4.88), as are the group differences in modulation (i.e. CON vs. PSP; *t* = −3.02, *p*_unc_ = 0.01, BF_10_ = 5.08). Moreover, with controlling for evoked power in the same sliding window (0–250 ms), we find that our group effects in beta1 modulation to remain significant (CON vs. PSP; *t* = −3.02, *p*_unc_ = 0.005), as are the patient group baseline differences in power (bvFTD vs. PSP; *t* = −2.69, *p*_unc_ = 0.01).

### 3.4 Association of beta1 power with cortical atrophy

**Fig. 3:**
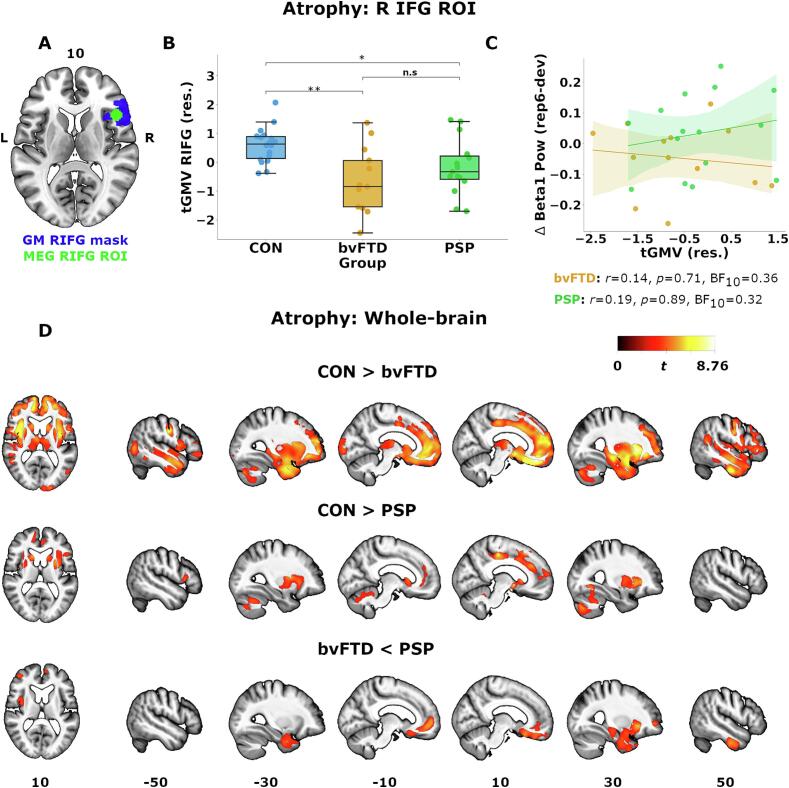
Group differences in cortical atrophy and association with beta1 modulation. A) Anatomical mask (blue areas) representing right inferior frontal gyrus (R IFG) region used for calculating GM volume, and its overlap with the magnetoencephalography (MEG) source region-of-interest (ROI; green sphere) used in time–frequency estimation. The anatomical mask is available at https://neurovault.org/images/776918. B) Distribution of residual GM volume scores in the R IFG region (adjusted for age and total intracranial volume) across groups. Boxes represents group mean with interquartile range and whiskers indicating 95 % probability density. C) Association between residual GM volume and condition-dependent beta1 modulation in each patient group (bvFTD, orange; PSP, green). D) Whole-brain group-wise voxel-based morphometry comparisons between controls and patient groups (cluster-level, *p* < 0.05, FWE-corrected; height-threshold, *p* < 0.001, uncorrected). Note, no significant cluster was found for bvFTD>PSP comparisons. n.s, non-significant; *, *p*_sidak_ < 0.05, corrected; **, *p*_sidak_ < 0.01, corrected. (For interpretation of the references to colour in this figure legend, the reader is referred to the web version of this article.)

In the right *a priori* prefrontal cortical area of interest (Fig. 3A), PSP caused modest atrophy (adjusting for age, sex, intracranial volume; *t* = −2.69, *p*_sidak_ = 0.027, BF_10_ = 5.30) while bvFTD caused severe atrophy relative to controls (*t* = −2.89, *p*_sidak_ = 0.002, BF_10_ = 50.72) (Fig. 3B). There was inconclusive evidence for a group difference between bvFTD and PSP (*t* = −0.30, *p*_sidak_ = 0.602, BF_10_ = 0.57). Whole-brain (grey-matter) VBM comparisons confirm significant atrophy in bvFTD in bilateral frontotemporal, striatal and thalamic areas, and atrophy in PSP in striatal, cerebellar, insular and medial frontal regions (Fig. 3D). bvFTD caused greater atrophy than PSP in ventromedial prefrontal cortex, and right insular and lateral prefrontal areas, while there were no regions with more severe atrophy in PSP than bvFTD (Fig. 3D).

There was no association between right prefrontal volume and beta power in either patient group (bvFTD: *r* = −0.14, *p*_unc_ = 0.71, BF_10_ = 0.36; PSP: *r* = 0.19, *p*_unc_ = 0.89, BF_10_ = 0.32), or when pooling groups together (*r* = 0.11, *p*_unc_ = 0.80, BF_10_ = 0.24) (Fig. 3C).

### 3.5 Association of beta1 power with clinical severity

**Fig. 4:**
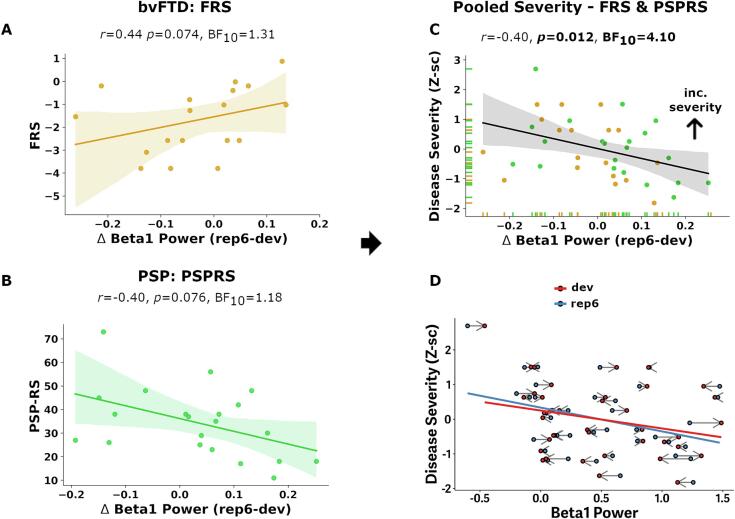
Association between beta1 power modulation and clinical severity. For bvFTD (A) and PSP (B) groups separately, association between beta1 power modulation and representative clinical severity measure. For bvFTD, the Frontotemporal Rating-Scale (FRS; log-transformed) and PSP the PSP Rating-Scale (PSP-RS). C) Scalar transformations (Z-scored) of each patient severity scores are combined into a pooled disease-severity score across groups, and its association with beta1 power modulation is shown. Rug plots on x- and y-axis indicate distribution of scores in each patient group for beta1 power modulation and pooled disease severity, respectively. D) Beta1 power as a function of trial-condition (*dev*, red line and circles; *rep6*, blue) and disease severity across patient groups, with the length and direction of arrows indicating within-subject differences in beta1 power responses across rep6 and dev trials. FRS, Frontotemporal Dementia Rating Scale; PSPRS, PSP Rating Scale. (For interpretation of the references to colour in this figure legend, the reader is referred to the web version of this article.)

While we find a trend association for clinical severity measures with beta1 power change in patient groups when analysed separately (FRS: *r* = 0.44*p* = 0.074, BF_10_ = 1.31; PSPRS: *r* = −0.40, *p* = 0.076, BF_10_ = 1.18; Fig. 4A and B), pooling normalized severity scores indicated a significant correlation (*r* = −0.40, *p* = 0.012, BF_10_ = 4.10; Fig. 4C). Inspecting the association of *rep6* and *dev* beta1 power separately with clinical severity, reveals the expected association of greater beta1 power in less severe patients. It also indicates that less severe patients express positive beta power changes (i.e. reduced *dev* response and greater beta response to rep6), which is lost as severity of disease increases (Fig. 4D).

Across each disease group and when pooled together, we found no association between beta power change and any of the individual cognitive measures (SI Table 4).

## 4 Discussion

There are three principal results from this study. First, that the mismatch negativity paradigm induces changes in beta power generated by the prefrontal cortex, which is moderated by the syndromes associated with frontotemporal lobar degeneration. Second, that there are differences in the effect of bvFTD and PSP, such that PSP reversed the mismatch negativity response (compared to controls), expressing increased beta1 responses to expected events (relative to deviants). Thirdly, changes in prefrontal beta1 power are proportional to disease severity. These changes in beta1 power are not simply attributable to atrophy, but may instead reflect the marked reductions in synaptic density and major neurotransmitters caused by PSP and bvFTD. Taken together with previous findings, the current results provide evidence to support our hypothesis of impaired beta-range function in FTLD-associated disorders.

Our findings of abnormal beta band dynamics in prefrontal cortex builds upon the evoked mismatch responses observed in frontotemporal lobar degeneration syndromes. Classic evoked mismatch signals are normally generated as result of transient dynamics within cortical units that are critical for generating beta signals (Adams et al., 2020, Ardid et al., 2015, Jones et al., 2009, Voloh and Womelsdorf, 2018). Such frequency-specific signals are propagated from deep pyramidal neurons in prefrontal cortex to lower-level regions (Bastos et al., 2020, Bastos et al., 2012, Michalareas et al., 2016), encoding sensory changes and signalling context-updating (Chao et al., 2018, Meindertsma et al., 2018, Sedley et al., 2016). Under a predictive coding account (one of several alternative accounts of the mismatch response) (Bastos et al., 2012, Friston, 2005), beta-mediated feedback signals during the oddball paradigm encode predictions of the expected incoming stimulus (i.e. the expected frequency after a series of auditory tones). Following this, the classic mismatch ERPs observed are reflective of prediction errors and their precision, propagated by predominantly gamma forward signalling (Hauke et al., 2023, Weber et al., 2020), and generated by a deviant of different frequency violating what was predicted via beta signals (Friston et al., 2016). In frontotemporal lobar degeneration, the failure of these beta feedback signals (Adams et al., 2021, Cope et al., 2022, Hughes and Rowe, 2013) to generate expectations of incoming sensory stimuli, may be one potential mechanism leading to the classically impaired error-detection signals (i.e. in response to deviant events). Here, the current findings of abnormal beta modulation further demonstrate the impaired ability of prefrontal areas to generate beta power in these syndromes. Beta dysfunction is not isolated to this paradigm, with impairments also in this frequency-band at rest (Sami et al., 2018) and during response inhibition (Hughes et al., 2018). Local microcircuit dynamics are contingent on disease state; with depletion of GABA (critical for beta-mediated signalling) and glutamatergic function (Adams et al., 2021, Perry et al., 2022), and also synaptic loss (Adams et al., 2022), being prime candidates for underpinning these neurophysiological changes.

We do not observe significant group differences in evoked mismatch negativity responses (i.e. MMN) to either bvFTD or PSP. This differs from previous studies (Cope et al., 2022, Hughes et al., 2013, Hughes and Rowe, 2013). The total beta power response is however distinct from evoked response amplitudes: the time–frequency estimates capture processes which are non-phase locked (as well as phase-locked in total power estimates) to the tone presentation, and hence optimal for capturing cognitive updating processes. Averaging trial responses as with both the mismatch negativity and evoked time–frequency calculations, removes such non phase-locked processes (Cohen, 2014, Tallon-Baudry et al., 1997). Such variations in the degree of evoked group differences from previous findings may be attributable to this methodological distinction, or to variations in the mismatch negativity paradigm, with deviant type and its temporal dynamics influencing change detection (Phillips et al., 2015), or clinical heterogeneity in each disease group (Murley et al., 2020a).

The significant group differences (relative to controls) in beta modulation were seen only in PSP. This begs the question of the unique disease pathologies contributing to this effect? bvFTD and PSP share cognitive deficits as well as the aforementioned neurotransmitter depletion (Murley et al., 2020a, Murley et al., 2020b, Murley and Rowe, 2018). Both diseases have also demonstrated deficits across cortical cell layers such as pyramidal cells and interneurons (Dickson et al., 2007, Levenga et al., 2013, Procter et al., 1999). Yet, in contrast to bvFTD, PSP shows a high clinicopathological correlation to four-repeat (4R)-tauopathy (Roesler et al., 2019), and also relatively mild cortical atrophy (Ghosh et al., 2012). It is possible that interactions between disease neuropathology (i.e. neurotransmitter decline, synaptic loss) and subtle cortical changes in PSP (observed here in whole-brain atrophy maps) leads to their unique prefrontal beta abnormalities. Indeed, synaptic loss, precipitated by tau disease (Kaniyappan et al., 2017), is found in both PSP and cortico-basal syndrome (CBS) (Holland et al., 2023, Holland et al., 2020, Kaniyappan et al., 2017, Lipton et al., 2001). However, from the current data, we can only speculate what these mechanisms are, guided by independent data on the pathologies of these disease groups; while bvFTD has higher cell loss and prefrontal atrophy (Ghosh et al., 2012, Kim et al., 2012), PSP causes severe synaptic loss, particularly in superficial cortical layers (Adams et al., 2022, Bigio et al., 2001, Holland et al., 2020). Further demonstrating a potential link between group differences here in PSP and synaptic loss, is that individual variation in synaptic density in PSP (estimated by ^11^C-UCB-J PET) is coupled to differences in neurophysiological function in the same prefrontal region used in the present study (Adams et al., 2022). Inversion of MEG data also with biophysical models could potentially illuminate the synaptic changes underpinning deficits to beta functioning here in PSP. The failure of bvFTD to modulate beta in either direction (with moderate evidence for no condition difference), which may be due to marked atrophy and neurotransmitter (Murley et al., 2020b, Perry et al., 2022) decline in this disease group.

An additional explanation for the unique PSP changes is suggested from studies of Parkinson’s Disease (PD). Like PSP, PD is also a neurodegenerative disorder characterised by beta abnormalities and similar clinical manifestations (in early stages) (Alster et al., 2020). Transient beta activity is generated from interactions between neocortex and basal ganglia/thalamic sites (Holgado et al., 2010, Sherman et al., 2016), and disruptions to these circuits (particularly subthalamic routes) are central to disease models of PD (Boeve et al., 2022, McCarthy et al., 2011, Moran et al., 2011, Oswal et al., 2021, Rae et al., 2016). From post-mortem studies, subcortical and brainstem nuclei are proposed as earliest sites for neuronal tau accumulation in PSP (Eser et al., 2018, Kovacs et al., 2020). Focal patterns of atrophy (relative to controls) in the brainstem and basal ganglia are consistent in PSP (Grossman et al., 2023) (and see Fig. 4D), and their neurophysiology are a key predictor of survival (Street et al., 2023, Whiteside et al., 2023). Thus, subcortical neuropathology may be a potential generator of abnormal beta-band neurophysiology in PSP.

Beta abnormalities are seen even at rest in PD. Beta modulation is critical for transition from task-free states to perception and goal-directed behaviours (Engel and Fries, 2010, Jones et al., 2010, Neuper et al., 2006). In PD, abnormally increased beta power and beta-burst dynamics in the basal ganglia (i.e. subthalamic nucleus) impairs shifting towards motor actions and goal-directed behaviours (Brown and Marsden, 1999, Neumann et al., 2016, Oswal et al., 2021, Tinkhauser et al., 2017), and notably specific to low beta ranges (Lofredi et al., 2019). Indeed, deficits in induced beta-responses in bvFTD impairs response inhibition (Hughes et al., 2018). Our post-hoc analyses of increased baseline beta1 power in PSP (relative to bvFTD) and gradual increases in beta1 power with tone-repetitions (contrary to progressive decrease in controls), is potentially indicative of abnormal beta-band dynamics in this disease group. Taken together, we can speculate that abnormally elevated beta1 power in PSP could impair task-related beta-modulation which mediates change detection and context signalling. Baseline activity (ending at the *peri*-stimulus onset) may be potentially influenced by evoked power, given the rapid-event design required with the roving paradigm. However, abnormal baseline gamma activity leading to deficits in oscillatory activity has been demonstrated also in Schizophrenia (Spencer, 2012), and our baseline differences were not between controls and PSP, and remain significant after controlling for individual differences in evoked power.

When pooling disease severity scores (PSP-RS and FRS) together, we observe a clinical correlation with beta1 modulation. The direction of effect may be initially unexpected: less clinically impaired persons show decreased deviant beta1 modulation (relative to expected trials), reflective of positive beta1 modulation. However, this reduction in beta responses needs to be seen in context, with both baseline states and temporal dynamics of beta1 activity (Jones, 2016). We find an expected negative association between disease severity and beta1 responses for both expected (*rep6*) and deviant trials. This suggests relatively intact neural activity (at least for *rep6* trials) in less clinically impaired patients. Moreover, abnormal beta synchrony in PD has been proposed as potential compensatory mechanism (Moran et al., 2011, Sparks et al., 2022), and following this thought we can speculate increased beta1 activity here could serve as an initial compensatory process which is lost as disease progresses. However, we cannot robustly test this association with disease duration in the current study; in FTLD, disease onset dates are often difficult to estimate, and pathological changes in genetic cases are observed in imaging or fluid biomarkers 10–15 years prior to symptom onset (Staffaroni et al., 2022). These interpretations generate further hypotheses that would require additional studies to resolve. Nonetheless, our findings are consistent with associations in frontotemporal lobar degeneration between induced beta activity and disinhibition (Hughes et al., 2018), and beta-mediated feedback signalling with cognitive performance (Adams et al., 2021). Lastly, considering our transdiagnostic clinical association, it points to a potential shared neuropathology in bvFTD and PSP underpinning abnormal beta-band activity. This encourages role of future studies linking beta power to synaptic density and neurotransmitter deficits*,* and pharmacological interventions targeting abnormal neurophysiology (Adams et al., 2021, Javitt et al., 2020, Murley et al., 2020b).

Mismatch negativity responses and its local cortical generators have been well-established for roving oddball paradigms (Fitzgerald and Todd, 2020, Garrido et al., 2009), and found to differ across many neurological and psychiatric conditions (i.e. Schizophrenia and Alzheimer’s Disease) (Erickson et al., 2016, Kocagoncu et al., 2021). Yet, most time–frequency studies in control populations have focused on (time-locked) evoked frequency responses, in contrast to the transient total power responses explored here. We do not find a significant condition difference for evoked beta1 responses, although the evidence (of no difference) is inconclusive. For these evoked responses, increases in lower-frequency activity reflected in alpha and theta bands (temporally overlapping with the mismatch response window, i.e. 100–250 ms) are consistently reported with deviant trials (Fuentemilla et al., 2008, Javitt et al., 2018, Lakatos et al., 2020, Lee et al., 2018). However, with slower-wave frequencies and short inter-trial intervals, it becomes difficult to estimate rapid-onset transient activity. An early study (Haenschel et al., 2000) of global induced EEG activity during the roving oddball paradigm revealed increased beta activity in response to deviants, and again specific to the beta1 range. Our non-significant exploratory based analyses (i.e. across the whole time–frequency search space) are likely attributable to effects being specific to the beta1 range, and the transient nature of the paradigm where beta activity peaks within the mismatch window. In the same study, increased induced gamma activity (to deviant trials) was coupled with beta1 changes, likely reflecting feedforward and feedback signalling respectively. Evidence for induced beta activity and higher-order regions (and coupled gamma activity) has largely come from intracranial recordings of other oddball paradigms (Sedley et al., 2016), such as the local–global paradigm (Axmacher et al., 2010, El Karoui et al., 2015). The present study extends the roving oddball literature finding increased beta modulation to be selective to right prefrontal cortex in the auditory network – consistent with higher-level regions such as the prefrontal cortex being generators of beta-mediated feedback signals. The rightward lateralisation is also interesting, and many studies of the generators of the mismatch response include right but not left prefrontal cortex (Garrido et al., 2009). As with the early studies applying DCM for the first time (Garrido et al., 2008, Garrido et al., 2007), our approach to estimating total (but not evoked) power to the mismatch negativity paradigm is also one of the first of its kind. Here we welcome further replication of time–frequency responses in independent and younger controls.

Here, we focused on beta responses overlapping with the canonical mismatch response temporal window (i.e. MMN) during the roving oddball paradigm. In contrast to late P300+ responses, the early neural processes related to change detection in this type of task are pre-attentive. There are limited effects of attentional modulation on these early components, and we note the preserved feedback signalling (and by extension beta-mediated signalling) of right prefrontal cortex even in minimally conscious patients (Boly et al., 2011). Our choice of passive watching reduces attention to the auditory domain, as has been recommended (Näätänen, 2000). It also helps to maintain arousal. Thus, individual differences in early beta activity here are not likely to be attributable to attentional differences. We did not employ an explicit visual distractor task as has been used in other roving oddball studies of less impaired populations (Garrido et al., 2008, Weber et al., 2020). A question for future research would be the frequency-specific effects across the auditory network during later cognitive processes such as the canonical P300 (Polich, 2007). A longer stimulus-onset-asynchrony, and a visual distractor task as in classic evoked oddball studies, would be advantageous for that purpose, to distinguish later frequency activity underlying stimulus-driven attentional changes, memory (Polich, 2007) and other higher-order representations (i.e. late prediction errors) (Weber et al., 2020).

There are limitations to this study. First, we focused solely on local power, and are agnostic to the influence of regional interactions across the auditory network. The short inter-trial interval also precluded estimation of lower-frequencies including theta and alpha-bands which are classically modulated in evoked oddball studies (Fuentemilla et al., 2008, Javitt et al., 2018, Lee et al., 2018). Moreover, by focusing on beta dynamics, we cannot rule out the contribution of alpha-band harmonics (Schaworonkow, 2023) and also cross-frequency interactions (i.e. gamma) generated from distinct laminar sites (Bastos et al., 2012, Haenschel et al., 2000). However, we focused on beta-band dynamics given the unequivocal evidence for its generation in higher-order areas, and the clear prefrontal abnormalities in frontotemporal lobar degeneration. Secondly, our diagnoses of PSP and bvFTD were made on clinical observations. Although the clinicopathological correlations are very high for both PSP (Pansuwan et al., 2023) and bvFTD, post-mortem diagnoses are required in further work to distinguish between tau versus *TDP43* pathology in bvFTD. However, our diagnoses of probable bvFTD and PSP are made with the high clinical confidence according to international consensus criteria, and the clinico-pathological accuracy is high and very high for bvFTD (Rascovsky et al., 2011) and PSP (Gazzina et al., 2019, Pansuwan et al., 2023) respectively*.* Further work is also required to examine the mechanisms underlying the disease differences observed in PSP. For instance, neurotransmitter decline through drug-studies and magnetic resonance spectroscopy (MRS) (Hirata et al., 2023), and synaptic loss via positron emission tomography (i.e. UCBJ). Thirdly, a small subset of patients (*n* = 10) did not have 7T imaging available (only 3T) and were not included in the atrophy analysis. Whilst it is common now to mix scans from separate scanners with harmonization approaches such as COMBAT, the different field strengths and sequences have markedly different segmentation performance. Moreover, including these patients would necessitate an independent control population also acquired on a 3T scanner, to calculate volume adjusted for age and sex. Lastly, there are statistical power considerations here with only *n* ∼ 20 per group, and which could account for the non-significant clinical correlations in the single disease groups. Early EEG oddball studies (Boly et al., 2011, Garrido et al., 2008) studied sample sizes of similar numbers (*n* = 21 patients and *n* = 12 controls, respectively). As with these studies, we hope that our findings motivate replication studies of beta modulation in independent control samples, and examination of the mechanisms of individual difference in larger clinical cohorts*.* Nonetheless, there was sufficient precision to draw statistical inferences, with Bayesian inference revealing positive or moderate-to-strong evidence for main effects of condition-dependent differences (BF_10_ = 6.54), and strong evidence for its group differences (CN vs. PSP, BF_10_ = 9.38).

With time–frequency studies it is critical to minimise the potential impact of physiological and external noise contaminants. These participant-level artifacts are exemplified by, but are not limited to, heart beats, eye movement and muscular-related activity (Muthukumaraswamy, 2013, Urigüen and Garcia-Zapirain, 2015). We chose an automatic ICA detection approach using EOG reference channels only to be consistent with our previous DCM (Adams et al., 2022) and time–frequency studies (Cope et al., 2022, Hughes et al., 2024, Hughes et al., 2018) in these disease groups. We could not additionally include ECG recordings to the tolerance for acquiring such recordings in these behaviourally challenging patients. While there is potential for also muscle-related artifacts (from both tonic and phasic sources), the influence is mitigated by further trial artifact detection (post ICA), our transient task design, and the focus on the low beta range (Muthukumaraswamy, 2013). Eye movements are not expected to drive our group differences, owing to the reduced ocular activity and eye blanks (c.f. SI Table 3) that are characteristic of PSP (Bologna et al., 2009).

In conclusion, we provide evidence for prefrontal beta-power neurophysiological abnormalities in frontotemporal lobar degeneration. We attribute the effects observed in PSP to disease-specific pathology, in keeping with the evidence for specific neurophysiological consequences across major neurodegenerative disorders (Ranasinghe et al., 2022, Sami et al., 2018, Samudra et al., 2024). Of relevance to normative systems neuroscience, is the influence here of (abnormal) local cortical dynamics on its transient non-“rhythmic” frequency-band activity, and its potential influence on large-scale brain activity. We suggest that changes in beta activity resulting from frontotemporal lobar degeneration may be an intermediate target for experimental studies.

## CRediT authorship contribution statement

**Alistair Perry:** Writing – review & editing, Writing – original draft, Visualization, Software, Methodology, Investigation, Formal analysis, Data curation, Conceptualization. **Laura E. Hughes:** Writing – review & editing, Project administration, Data curation, Conceptualization. **Natalie E. Adams:** Writing – review & editing, Project administration, Methodology, Data curation. **Michelle Naessens:** Writing – review & editing, Project administration, Data curation. **Niels A. Kloosterman:** Writing – review & editing, Resources, Methodology. **Matthew A. Rouse:** Writing – review & editing, Data curation. **Alexander G. Murley:** Writing – review & editing, Resources, Methodology, Data curation. **Duncan Street:** Writing – review & editing, Data curation. **P. Simon Jones:** Writing – review & editing, Resources, Methodology. **James B. Rowe:** Writing – review & editing, Writing – original draft, Supervision, Project administration, Methodology, Investigation, Funding acquisition, Data curation, Conceptualization.

## Declaration of competing interest

The authors declare that they have no known competing financial interests or personal relationships that could have appeared to influence the work reported in this paper.

## Data Availability

The authors do not have permission to share data.
