# Supplementary material for: Frontotemporal lobar degeneration changes neuronal beta-frequency dynamics during the mismatch negativity response
Source: Neuroimage Clin. 2024 Sep 10;44:103671. doi: 10.1016/j.nicl.2024.103671 (PMC11439566; doi:10.1016/j.nicl.2024.103671)
Supplement: Supplementary Data 1 [file mmc1.docx]

**Supplementary Information, Figures and Tables:**

Frontotemporal lobar degeneration changes neuronal beta-frequency dynamics during the mismatch negativity response

Alistair Perry^1,2^(PhD), Laura E. Hughes^1,2^(PhD), Natalie Adams^2^(PhD), Michelle Naessens^1,2^(MSc), Niels A. Kloosterman^3,4^(PhD), Matthew A. Rouse^1^(PhD), Alexander G. Murley^2^(PhD), Duncan Street^2^(PhD), P Simon Jones^2^(MSc), James B. Rowe^1,2^(PhD)†

^1^ MRC Cognition and Brain Sciences Unit, University of Cambridge, Cambridge, UK

^2^ Department of Clinical Neurosciences and Cambridge University Hospitals NHS Trust, University of Cambridge, United Kingdom

^3^ Institut für Psychologie I, Universität zu Lübeck, Germany

^4^ Max Planck Institute for Human Development, Berlin, Germany

† Corresponding Author:

Professor James Rowe, Department of Clinical Neurosciences, University of Cambridge, Herchel Smith Building, Forvie Site, Robinson Way, Cambridge Biomedical Campus, Cambridge CB2 0SZ,

Email: [james.rowe@mrc-cbu.cam.ac.uk](mailto:james.rowe@mrc-cbu.cam.ac.uk)

**1. Supplementary Information**

1.1. *Auditory roving oddball paradigm:*

MEG was recorded while participants completed an auditory roving oddball paradigm (Adams et al., 2020; Garrido et al., 2008). Binaural sinusoidal tones (60dB above the population average threshold) were presented for 75ms to participants through headphones, with a 7.5ms ramp up and down at the start and end of the tone, and with stimulus onset asynchrony of 500ms (ITI). This roving oddball paradigm comprises mini-blocks of 3-10 tone repetitions, where at the end of each mini-block the tone frequency changed pseudorandomly. This first tone of each block changing in frequency is defined as the deviant (*dev*) tone. Tone frequencies presented were in the range of 400-800Hz. Participants were under continuous video monitoring to ensure none fell asleep, and they were not asked to attend to the auditory stimuli. The paradigm was performed eyes-open in blocks of five minutes while participants watched a movie (i.e. walking with dinosaurs), with an average number of 1669 (SD=151) stimulus trials (before trial rejection) across each subject and session.

1.2. *MEG data acquisition and preprocessing:*

MEG data were acquired in a magnetically-shielded (IMEDCO) room using the Elekta VectorView system (Elekta Neuromag, Helsinki). This MEG system comprises of 306-channel recordings at 102 spatial locations (a pair of planar gradiometers and a magnetometer at each site) and was sampled at 1000 Hz, with a high-pass filter of 0.03 Hz. Electroocculograms (EOGs) tracked eye movements vertically and horizontally and 5 head position indicator coils tracked head position every 200ms. A 70 channel, MEG-compatible, electroencephalogram (EEG) cap (Easycap GmbH) using Ag/AgCl electrodes positioned according to the 10-20 system was used concurrently, although this modality was not used for rejecting bad trials, source reconstruction, nor in subsequent analysis. Scalp shape was recorded with a 3D digitizer (Fastrak Polhemus Inc., Colchester, VA) using > 100 scalp points, as well as the position of the nasion and bilateral pre-auricular fiducial points. Auditory stimuli were delivered binaurally through MEG-compatible ER3A insert earphones (Etymotic Research). Instructions and the video were presented on the screen positioned 1.22 meters in front of the participant’s visual field.

To ensure that the earphones were working correctly, before the MEG recording participants performed an automated hearing test in the scanner. They were presented tones at 1000Hz to either ear with varying loudness, and instructed to press the button when they heard the tone.

Preprocessing of MEG data was performed in SPM12 (www.fil.ion.ucl.ac.uk/spm, v7771), FieldTrip (Oostenveld et al., 2011) and OSL (https://github.com/OHBA-analysis/osl-core) software packages in Matlab (2019a) (pipeline available at https://github.com/AlistairPerry/FTLDMEGMEM).

First, the raw E/MEG were preprocessed using MaxFilter 2.2.12 in Matlab 2018a (Elekta Neuromag, https://imaging.mrc-cbu.cam.ac.uk/meg/Maxfilter_V2.2). This included the following steps; Interpolation of bad channels, which were automatically detected using the default threshold (*r*=0.7, the number of bad channels detected across groups are reported in SI Table 2). Next, signal source separation was performed to remove noisy signals from outside the brain and lastly head motion correction. Next, the data were downsampled to 500Hz, band-pass filtered (0.1-125 Hz using a fifth-order Butterworth filter) and further notch filtered to remove 50 (45-55Hz) and 100 Hz (95-105Hz) line noise (Kocagoncu et al., 2022). Artefact rejection (*osl_detect_artefacts)* was first used to remove bad channels, and then independent component analysis (ICA) further removed eye-movement related artefacts. ICA involved: A fast fixed-point algorithm, 800 maximum steps, 60 principal components, symmetric approach, tan-h non-linearity, epsilon of 0.00001, via the *FastICA* package for MATLAB. The independent component time series were correlated with the VEOG and HEOG channel time series. The components that revealed correlations higher than *r* = 0.35 were removed and the data of the remaining independent components were reconstructed.

The continuous MEG data were then epoched into separate temporal windows: (1) single-trials padded to include both the preceding and proceeding trial of each event-of-interest (-750 to 1050ms), and (2) epoched in the original range (-100 to 400ms) for analysis of evoked mismatch waveforms. Both epoching approaches included the removal of bad trials using OSL’s artefact rejection (using magnetometers and gradiometers). The non-padded data were averaged using robust averaging and then again low-pass filter corrected (125 Hz) to remove high-frequency noise induced after averaging.

Artifact MEG channels and trials were both removed using *osl_detect_artefacts*, which identifies outliers through a Generalized Extreme Studentized Deviate (ESD) test, at the default significant threshold of α=0.05.

1.3. *Acquisition parameters and preprocessing of T1-weighted structural imaging scan*

Subjects completed either a 7T T_1_-weighted structural scan on a Siemens TERRA scanner (Siemens Healthineers, Erlangen, Germany) (Nova Medical, Massachusetts, USA), or a 3T Siemens PRISMA scanner. The acquisition parameters of the 7T MP2RAGE sequence included: 0.75mm isotropic voxels, TE=1.99ms, TR=4300ms, resolution = 99ms, bandwidth = 250 Hz/px, voxel size = 0.75 mm^3^, field of view = 240 × 240 × 157 mm, acceleration factor (A ≫ P) = 3, flip-angle = 5/6° and inversion times = 840/2370ms). The acquisition of the 3T MPRAGE sequence included: TE = 2.9ms, TR = 2000ms, 1.1 mm isotropic voxels.

Only participants with 7T MP2RAGE sequence were included in the analysis of prefrontal cortical atrophy (for association with beta-frequency responses) and whole-brain atrophy patterns.

For these individuals, we first performed signal bias correction (O'Brien et al., 2014) and segmentation of grey matter (GM), white matter (WM), and cerebrospinal fluid (CSF) segments using the standard settings in SPM12 (v7771). Next, we created a study-specific template image with differomorphic registration (DARTEL) (Ashburner, 2007) of native space grey and white matter images from an equal number of control, bvFTD and PSP individuals. Each participant's native GM image were then normalised to standard space by applying the deformation combined with the affine transformation parameters (i.e. native-space to group average to MNI space) which included modulation in order to preserve local volume. The images were lastly smoothed with a Gaussian kernel at 8 mm full width half maximum (FWHM). The total intracranial volume (TIV) for each participant was calculated using the Tissue Volumes function in SPM12.

Grey matter volume (GMV) in the right inferior-frontal gyrus region-of-interest was calculated from the total sum of voxel probabilities within a pre-defined anatomical mask (for further details see (Perry et al., 2022)) enclosing the individual normalized grey-matter images. The normalization included modulation in order to preserve local volume, but without smoothing.

*1.4 Voxel-Based Morphometry*

Voxel-based morphometry (VBM) was performed used in SPM12 to compare whole-brain GM volume between controls and across the diagnostic groups. GM volumes for each diagnostic group were compared with independent two-sample *t*-tests with age, sex and total intracranial volume used as covariates of no interest (Barnes et al., 2010). Significant effects were identified using cluster-level statistics (*p*<0.05, family-wise error corrected for multiple comparisons) above a height threshold of *p*<0.001 (uncorrected).

**2. Supplementary Figures**

**
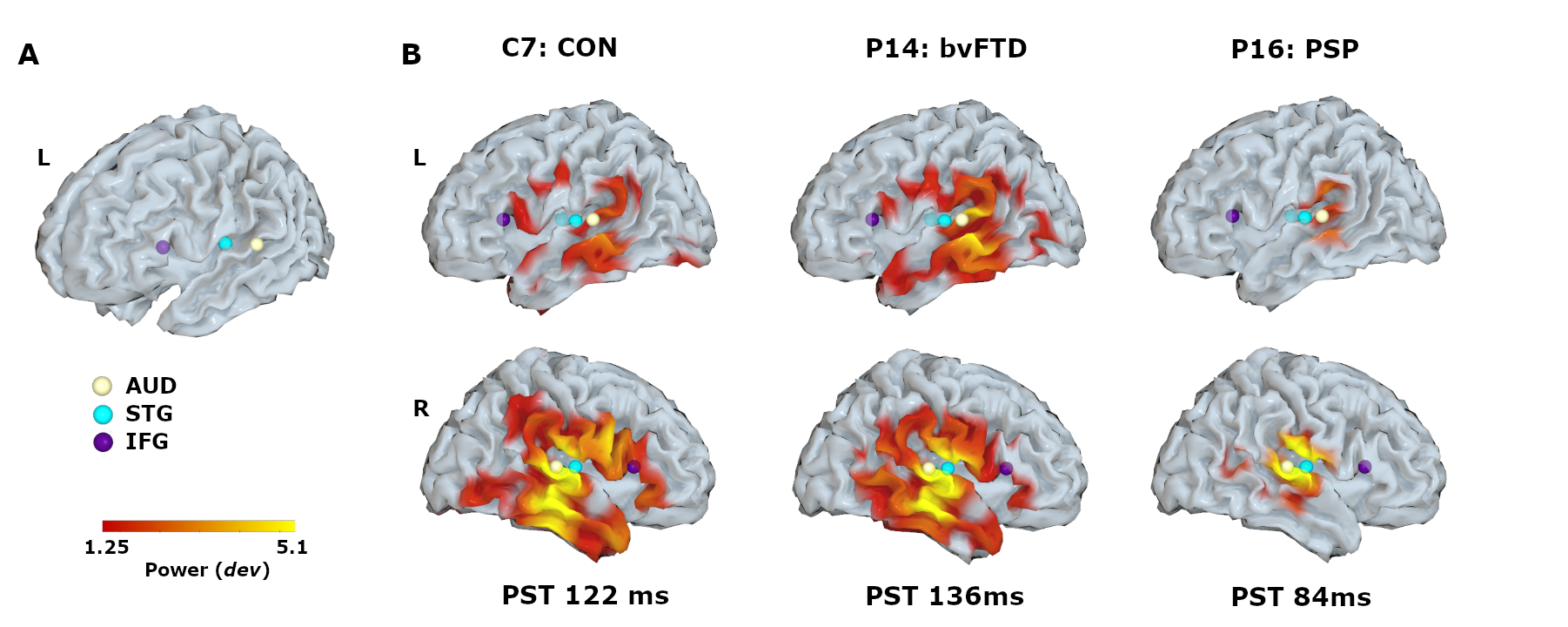
**

**SI Fig 1. Spatial correspondence between literature-derived MNI coordinates of mismatch negativity sources and source reconstruction results.** A) MNI locations (left hemisphere only) of mismatch negativity sources used in magnetoencephalography analysis, with sphere colour indicating the anatomical region. Note, sphere size is not representative of mask radius used for extracting LFP signal responses. B) Exemplar surface source reconstruction results of deviant (*dev*) evoked condition responses using COH inversion for exemplar individuals from each study group. Source images are extracted at the peristimulus time (PST) of maximal activation in each individual, with heatmap representing magnitude of power at that timepoint. Note, a supra-threshold heatmap value of 1.25 has been used.

IFG, Left Inferior Frontal Gyrus; STG, Left Superior Temporal Gyrus; AUD, Auditory Cortex; L, Left Hemisphere; R, Right Hemisphere

**
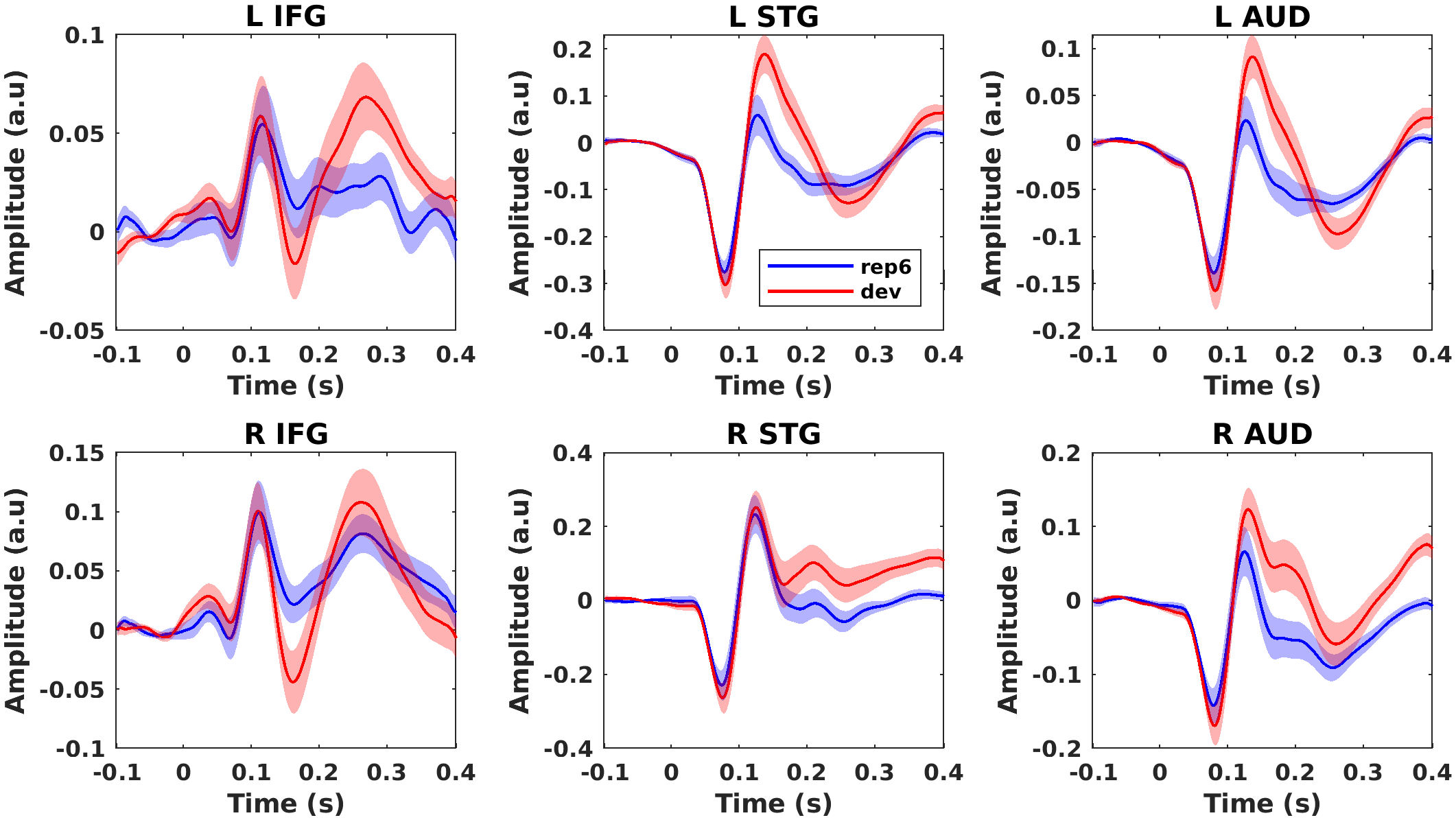
**

**SI Fig 2. In controls, group averaged evoked waveforms of single-condition *dev* (red line and shading) and *rep6* (blue) responses across frontotemporal source regions.** Solid lines represent the group average with the shading indicating standard error at each time point.

L IFG, Left Inferior Frontal Gyrus; L STG, Left Superior Temporal Gyrus; L AUD, Left Auditory Cortex; R IFG, Right Inferior Frontal Gyrus; R STG, Right Superior Temporal Gyrus; R AUD, Right Auditory Cortex


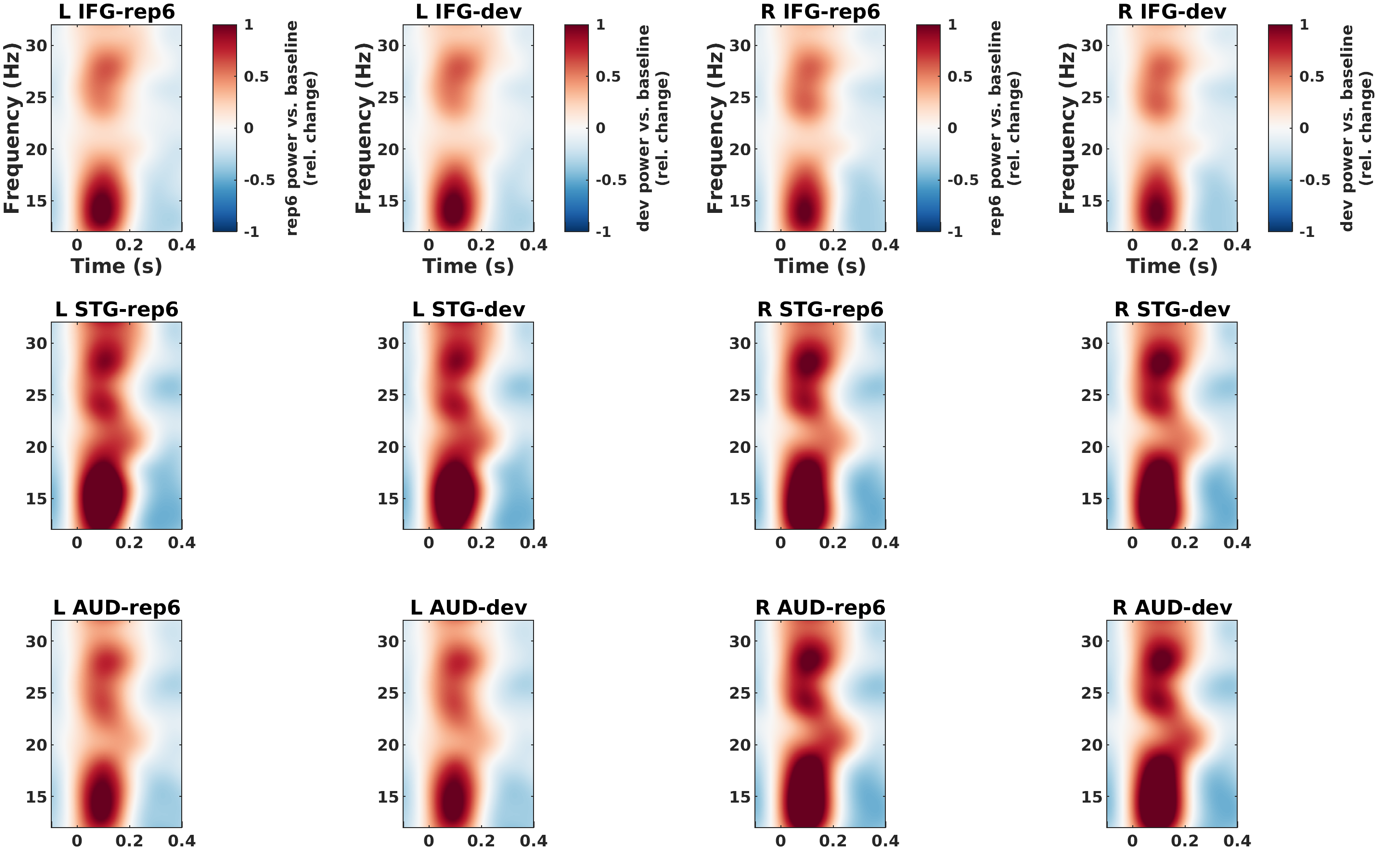


**SI Fig 3. Group average single-condition time-frequency responses in controls for *rep6* and *dev* trials across frontotemporal sources**. Heatmap at each time point for each frequency bin indicates magnitude and direction of power modulation, expressed as relative change compared to the baseline period (-100 to 0ms) for its respective frequency bin. For each source region, the *rep6* condition is shown on the left panel, while *dev* trials are on the right. Note, heatmaps values represent interpolated responses.

L IFG, Left Inferior Frontal Gyrus; L STG, Left Superior Temporal Gyrus; L AUD, Left Auditory Cortex; R IFG, Right Inferior Frontal Gyrus; R STG, Right Superior Temporal Gyrus; R AUD, Right Auditory Cortex


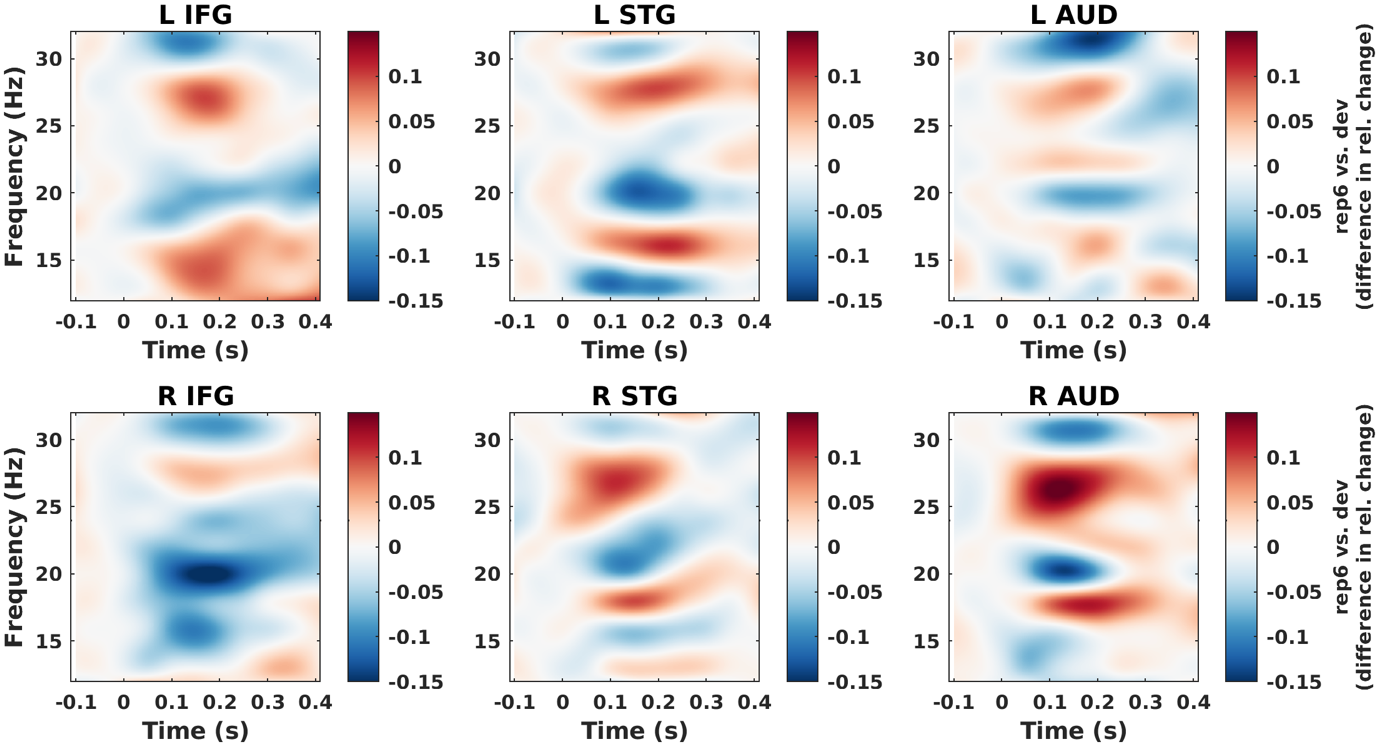


**SI Fig 4. In controls, group average differences in time-frequency responses between *rep6* and *dev* trial-conditions across frontotemporal sources**. Heatmap at each time point for each frequency bin indicates condition-differences in magnitude and direction of power (i.e. differences in relative change to baseline across conditions). Note, heatmaps represent interpolated difference responses.

L IFG, Left Inferior Frontal Gyrus; L STG, Left Superior Temporal Gyrus; L AUD, Left Auditory Cortex; R IFG, Right Inferior Frontal Gyrus; R STG, Right Superior Temporal Gyrus; R AUD, Right Auditory Cortex


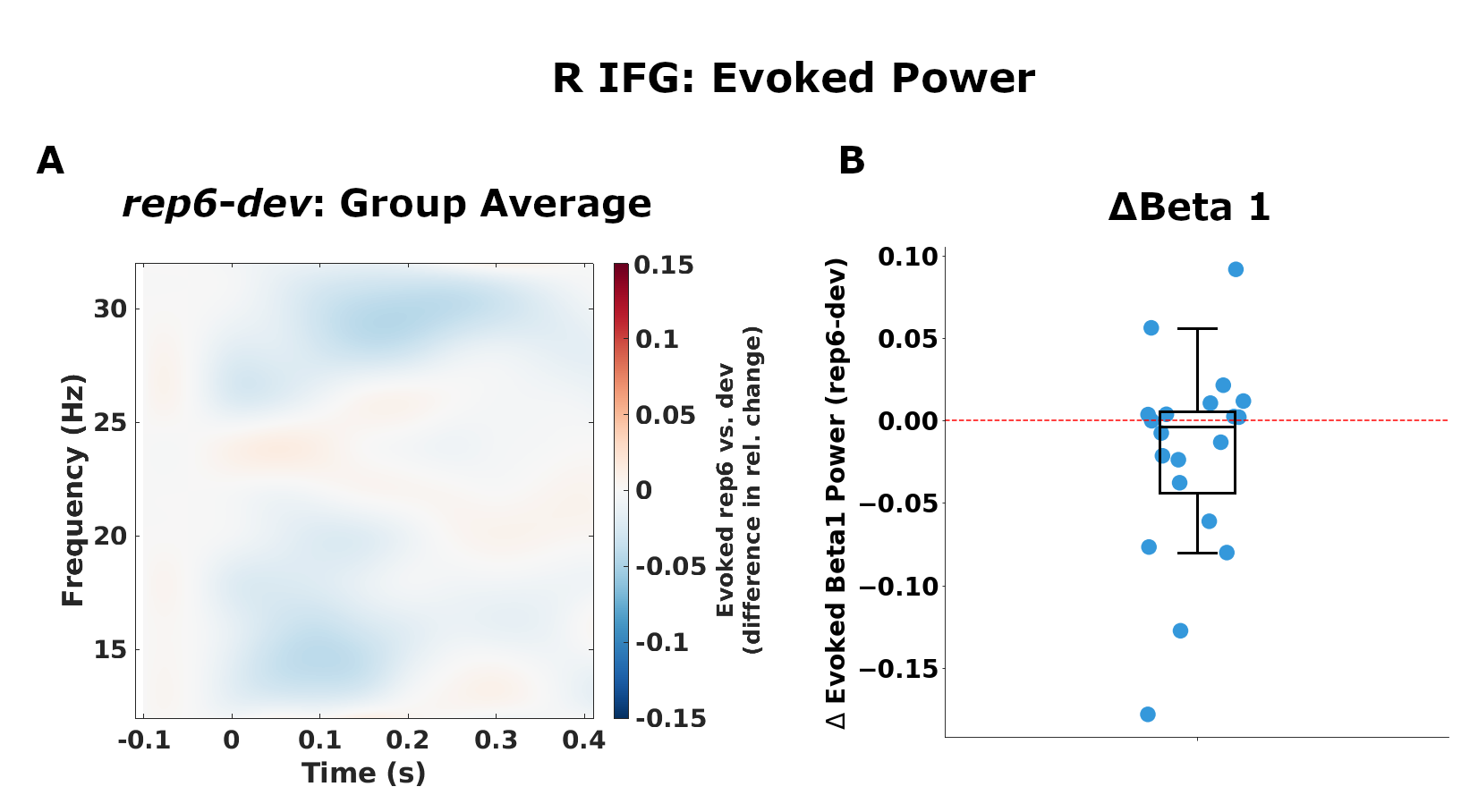


**SI Fig 5. In controls, evoked power differences in time-frequency responses between *rep6* and *dev* trial-conditions in the right inferior frontal gyrus (R IFG).** A) In controls, group-average condition differences across the entire time-frequency window estimated. Heatmap at each time point within each frequency bin indicates condition-differences in magnitude and direction of power (i.e. differences in relative change compared to their baseline). Note, heatmap values represent interpolated difference responses, and colour bar is scaled according to group-average total power (c.f. Fig 1B and 2A). B) Distribution of each control participants condition-dependent changes in evoked beta1 power. Box plot represents group mean with interquartile range, and whiskers indicating 95% probability density.

**
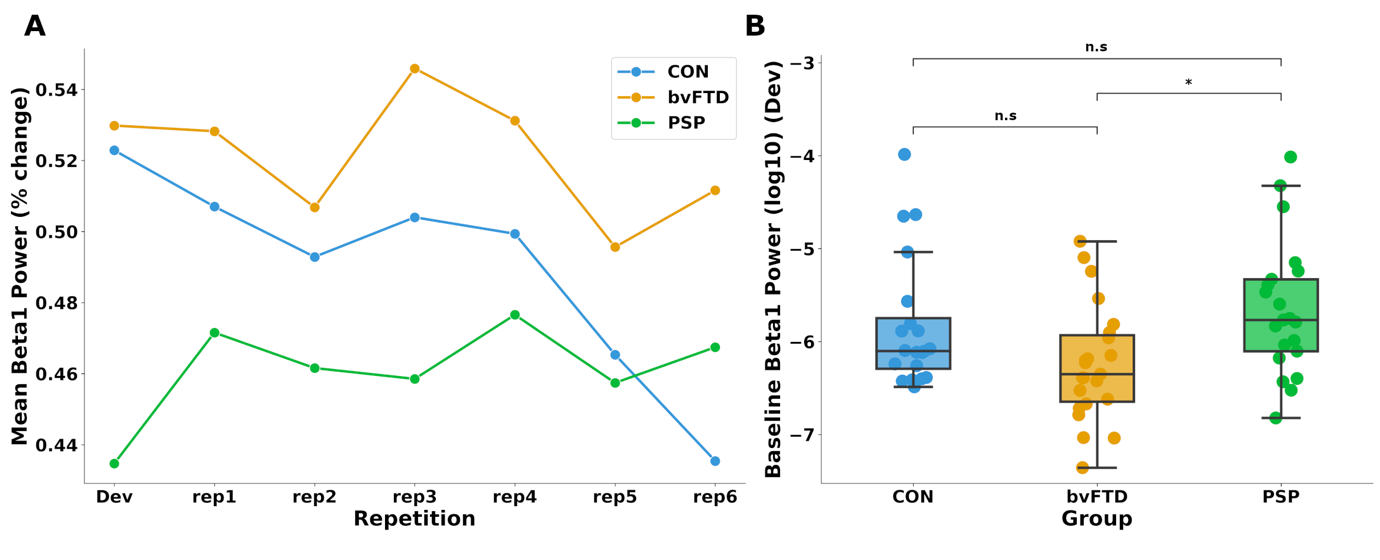
**

**SI Fig 6. Dynamics of beta1 responses in the right inferior frontal source region across groups during the roving oddball paradigm**. A) Dynamics of beta1 power responses (relative change compared to the baseline) as a function of trial repetition, with data points indicating the group average at each repetition. B) Group differences in baseline beta1 power (log-transformed) during the baseline period (-100 to 100ms) of *dev* trials. Boxes represents group mean with interquartile range and whiskers indicating 95% probability density.

n.s, non-significant; *, *p*_sidak_ < 0.05, corrected.


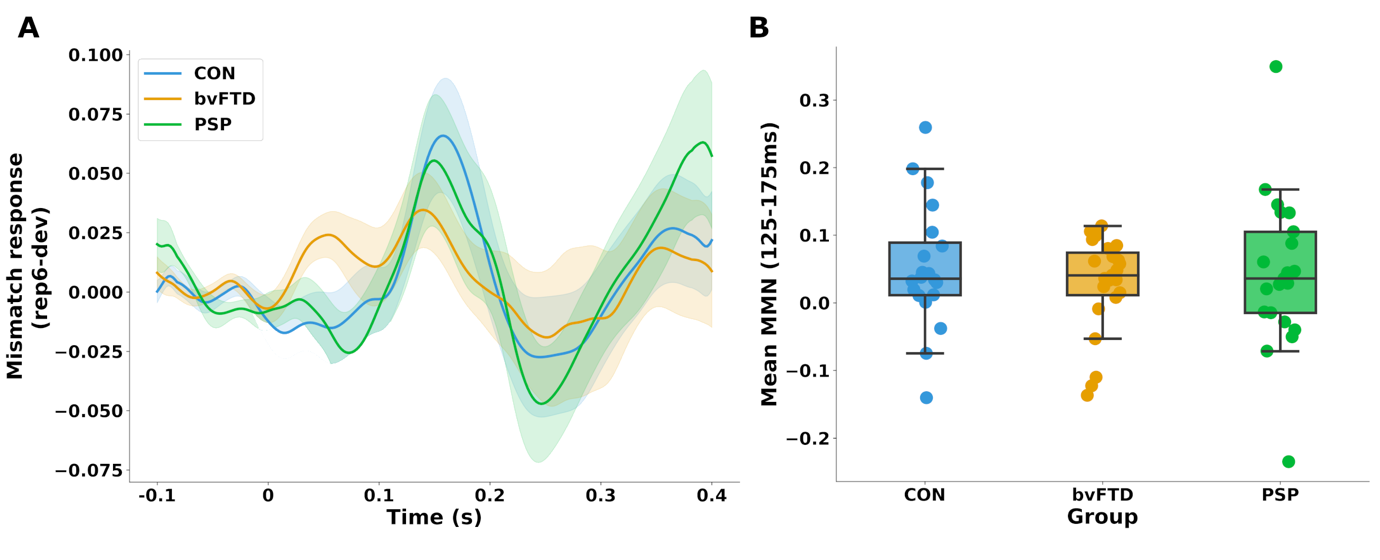


**SI Fig 7. Mismatch waveforms and mean mismatch negativity (MMN) responses.** A) Group average mismatch waveforms in right inferior frontal cortex across peri-stimulus window for controls (blue line), bvFTD (orange), and PSP (green) groups. Mismatch responses were derived from each individual by the difference of their *rep6* and *dev* waveforms. Thick lines and shading represent group average and its standard error at each time-point, respectively. B) Mean MMN responses (average of mismatch waveform between 125-175 ms) in controls and disease subgroups. Boxes represents group mean with interquartile range, and whiskers indicating 95% probability density. Note, no significant group differences were identified for mean MMN responses.

**3. Supplementary Tables**

**SI Table 1 – Number of bvFTD and PSP persons on medications during the study**

| **Medication** | **bvFTD** | **PSP** |
| --- | --- | --- |
| **Anti-depressant / SSRI’s** | 7 | 9 |
| **Benzodiazepines or Z-drugs** | 1 | 2 |
| **DA agonists or l-dopa*** | 1 | 7 |
| **Amantadine** | 2 | 6 |

Anti-depressant / SSRI’s; Citalopram, Trazadone, Fluoxetine, Sertraline, Mirtazapine, Amitriptyline

Benzodiazepines; Temazepam, Clonazepam, Diazepam

Z-drugs; Zopiclone, Zolpidem

DA agonists or l-dopa; Madopar, Sinemet (Cobeneldopa, Cocareldopa)

bvFTD, behavioural variant frontotemporal dementia; PSP, progressive supranuclear palsy

**SI Table 2 – Descriptive statistics for comparison of CBI-R total and subscales across bvFTD and PSP groups**

|  | **bvFTD** | **PSP** | **bvFTD vs. PSP** |
| --- | --- | --- | --- |
|  | **Mean (SD)** | | ***p*-val (BF_10_)** |
| Total | 89.9 (28.41) | 49.86 (30.06) | *** (376.38) |
| Disinhibition | 34.23 (14.98) | 11.76 (9.53) | *** (13.79e+3) |
| Memory and orientation | 16.52 (5.9) | 7 (5.47) | *** (9116.53) |
| Everyday skills | 9.52 (6.31) | 10.14 (7.54) | n.s (0.31) |
| Self-care | 4.52 (4.63) | 5.52 (5.48) | n.s (0.38) |
| Abnormal behaviour | 13.62 (6.24) | 3.43 (3.38) | *** (46.16e+3) |
| Mood | 5.9 (3.39) | 2.43 (2.36) | *** (63.37) |
| Beliefs | 1.86 (2.15) | 0.55 (1.15) | * (2.26) |
| Eating habits | 9.57 (5.28) | 3.95 (4.48) | *** (70.11) |
| Sleep | 3.62 (2.82) | 3.25 (2.34) | n.s (0.33) |
| Stereotypic and motor | 11 (5.39) | 4.38 (4.79) | *** (247.38) |
| Motivation | 14.14 (4.64) | 9.38 (6.09) | ** (7.57) |

CBI-R tests between bvFTD and PSP are uncorrected for multiple comparisons

**p*<0.05; ***p*<0.01; ****p*<0.001, uncorrected.

BF, Bayes Factor; Conventional thresholds for Bayes Factors represent substantial (>3), strong (>10) and very strong (>30) evidence in favour of alternate hypothesis.

CBI-R, Cambridge Behavioural Inventory Revised

**SI Table 3 – Quality assurance of MEG recordings and trials**

|  | **CON** | **bvFTD** | **PSP** |
| --- | --- | --- | --- |
| **Mean (SD)** | | | |
| ***MEG artefacts*** | | | |
| **Bad channels** | 8.25 (9.47) | 8.00 (4.06) | 8.74 (6.21) |
| **Eye blinks**  **(per minute)** | 10.67 (4.52) | 8.52 (5.23) | 5.49 (2.24) |
| ***ICA eye-components***  ***removed (n)*** | | | |
| **Temporal** | 3.40 (1.10) | 3.05 (1.99) | 1.00 (1.55) |
| **Spatial** | 5.70 (1.69) | 6.77 (3.01) | 5.95 (3.11) |
| ***MEG trials (n)*** | | | |
| **All trials** | 1578.75 (98.0) | 1639.30 (143.12) | 1680.57 (198.58) |
| **rep6** | 138.45 (7.89) | 142.96 (11.80) | 146.57 (18.18) |
| **dev** | 215.95 (13.74) | 223.22 (18.89) | 229.38 (25.89) |
| **% Bad trials** | 2.5 (3.3) | 2.6 (3.3) | 1.2 (1.3) |
| ***Group Contrasts*** | **CON vs. bvFTD** | **CON vs.**  **PSP** | **bvFTD vs.**  **PSP** |
| ***p*.val (BF_10_)** | | | |
| **Bad channels** | n.s (0.31) | n.s (0.31) | n.s (0.32) |
| **Eye blinks** | n.s (0.66) | *** (560.35) | * (3.03) |
| **Spatial ICA** | n.s (0.66) | n.s (0.32) | n.s (0.41) |
| **Temporal ICA†** | n.s (0.36) | *** (60.10) | *** (11.20) |
| **All trials**† | n.s (0.38) | n.s (0.44) | n.s (0.33) |
| **% Bad trials** | n.s (0.86) | n.s (1.17) | n.s (0.30) |

† Mann-Whitney U tests were used for both frequentist and Bayesian comparisons

BF, Bayes Factor; Conventional thresholds for Bayes Factors represent substantial (>3), strong (>10) and very strong (>30) evidence in favour of alternate hypothesis.

*, *p*<0.05; ***, *p*<0.001

**SI Table 4 – Association between beta power change and cognitive and clinical phenotypic scores**

| **Variable** | **bvFTD** | | **PSP** | | **bvFTD/PSP** | |
| --- | --- | --- | --- | --- | --- | --- |
|  | ***r*** | ***p* (BF_10_)** | ***r*** | ***p* (BF_10_)** | ***r*** | ***p* (BF_10_)** |
| **ACER** | -0.12 | n.s (0.29) | 0.24 | n.s (0.46) | 0.04 | n.s (0.19) |
| **FAB** | -0.03 | n.s (0.27) | 0.15 | n.s (0.33) | 0.08 | n.s (0.22) |
| **Disinhibition** | -0.03 | n.s (0.27) | -0.10 | n.s (0.30) | -0.22 | n.s (0.50) |

BF, Bayes Factor; Conventional thresholds for Bayes Factors represent substantial (>3), strong (>10) and very strong (>30) evidence in favour of alternate hypothesis.

ACE-R Addenbrooke's Cognitive Examination-Revised, FAB frontal assessment battery

**4. References**

Adams, N.E., Hughes, L.E., Phillips, H.N., Shaw, A.D., Murley, A.G., Nesbitt, D., Cope, T.E., Bevan-Jones, W.R., Passamonti, L., Rowe, J.B., 2020. GABA-ergic dynamics in human frontotemporal networks confirmed by pharmaco-magnetoencephalography. Journal of Neuroscience 40, 1640-1649.

Ashburner, J., 2007. A fast diffeomorphic image registration algorithm. Neuroimage 38, 95-113.

Barnes, J., Ridgway, G.R., Bartlett, J., Henley, S.M., Lehmann, M., Hobbs, N., Clarkson, M.J., MacManus, D.G., Ourselin, S., Fox, N.C., 2010. Head size, age and gender adjustment in MRI studies: a necessary nuisance? Neuroimage 53, 1244-1255.

Garrido, M.I., Friston, K.J., Kiebel, S.J., Stephan, K.E., Baldeweg, T., Kilner, J.M., 2008. The functional anatomy of the MMN: a DCM study of the roving paradigm. Neuroimage 42, 936-944.

Kocagoncu, E., Nesbitt, D., Emery, T., Hughes, L., Henson, R.N., Rowe, J.B., 2022. Neurophysiological and brain structural markers of cognitive frailty differ from Alzheimer’s disease. Journal of Neuroscience.

O'Brien, K.R., Kober, T., Hagmann, P., Maeder, P., Marques, J., Lazeyras, F., Krueger, G., Roche, A., 2014. Robust T1-weighted structural brain imaging and morphometry at 7T using MP2RAGE. PloS one 9, e99676.

Oostenveld, R., Fries, P., Maris, E., Schoffelen, J.-M., 2011. FieldTrip: open source software for advanced analysis of MEG, EEG, and invasive electrophysiological data. Computational intelligence and neuroscience 2011.

Perry, A., Hughes, L.E., Adams, N., Naessens, M., Murley, A.G., Rouse, M.A., Street, D., Jones, P.S., Cope, T.E., Kocagoncu, E., 2022. The neurophysiological effect of NMDA-R antagonism of frontotemporal lobar degeneration is conditional on individual GABA concentration. Translational psychiatry 12, 1-12.
